# The Role of p21-Activated Kinases in Cancer and Beyond: Where Are We Heading?

**DOI:** 10.3389/fcell.2021.641381

**Published:** 2021-03-16

**Authors:** Hui Liu, Kangdong Liu, Zigang Dong

**Affiliations:** ^1^Department of Pathophysiology, School of Basic Medical Sciences, The Academy of Medical Science, College of Medical, Zhengzhou University, Zhengzhou, China; ^2^China-US (Henan) Hormel Cancer Institute, Zhengzhou, China

**Keywords:** PAKs, cancer, disease, mechanism, inhibitor

## Abstract

The p21-activated kinases (PAKs), downstream effectors of Ras-related Rho GTPase Cdc42 and Rac, are serine/threonine kinases. Biologically, PAKs participate in various cellular processes, including growth, apoptosis, mitosis, immune response, motility, inflammation, and gene expression, making PAKs the nexus of several pathogenic and oncogenic signaling pathways. PAKs were proved to play critical roles in human diseases, including cancer, infectious diseases, neurological disorders, diabetes, pancreatic acinar diseases, and cardiac disorders. In this review, we systematically discuss the structure, function, alteration, and molecular mechanisms of PAKs that are involved in the pathogenic and oncogenic effects, as well as PAK inhibitors, which may be developed and deployed in cancer therapy, anti-viral infection, and other diseases. Furthermore, we highlight the critical questions of PAKs in future research, which provide an opportunity to offer input and guidance on new directions for PAKs in pathogenic, oncogenic, and drug discovery research.

## Introduction

Various protein kinases have been identified to be drivers in human cancer progression, and targeting oncogenic protein kinases has been developed successfully for cancer therapy. However, drug resistance is frequently occurring during the treatment processes. The p21-activated kinases (PAKs) are serine/threonine kinases and are the downstream effectors of Ras-related Rho GTPase Cdc42 and Rac (Morrice et al., 1994). There are six members in PAKs family, which are divided into two groups based on the sequence and structure homology: group I PAKs (including PAK1, PAK2, and PAK3) and group II PAKs (including PAK4, PAK5, and PAK6). Among all PAKs, PAK1, and PAK4 have been well studied, and other PAKs are getting researchers’ interest (Geng et al., 2020; Maruta and Kittaka, 2020; Møller et al., 2020; Ruan et al., 2020; Zheng et al., 2020). Most of the PAKs have oncogenic effects on cancer development. Moreover, alterations (including amplifications and mutations) of PAKs are frequently found in different cancers. Besides cancer, PAKs are critical in human neurological disorders, diabetes, pancreatic acinar diseases, and cardiac disorders (Li and Minden, 2003; Meng et al., 2005; Liu et al., 2011; Lee et al., 2019). PAK1, PAK2, and PAK4 are the major pathogenic-related kinases among all PAKs, whose abnormal activation is involved in inflammation (asthma and arthritis), malaria, and infection, including bacteria, virus [human immunodeficiency virus (HIV), influenza, and severe acute respiratory syndrome coronavirus 2 (SARS-CoV-2)], and protists, tuberous sclerosis, depression, schizophrenia, epilepsy, and autism beyond cancer (Chan and Manser, 2012; Maruta, 2014; Berretta et al., 2020). Thus, PAKs may serve as potential therapeutic targets for drug discovery in cancer and other diseases. Indeed, some small molecular compounds have been developed as PAK inhibitors for cancer restriction, infectious diseases, including SARS and SARS-CoV-2, mental retardation (MR), cardiac disorders, diabetes, and pancreatic acinar disease therapy (Maruta and He, 2020). Herein, we summarize the structure, function, alteration, and molecular mechanisms of different PAKs that involved in the cancer development, pathogenic progression, and infection. We also review PAK inhibitors (natural and synthetic) for their possible use in cancer therapy and treatment of other diseases.

## Structure and Activation of p21-Activated Kinases

**FIGURE 1:**
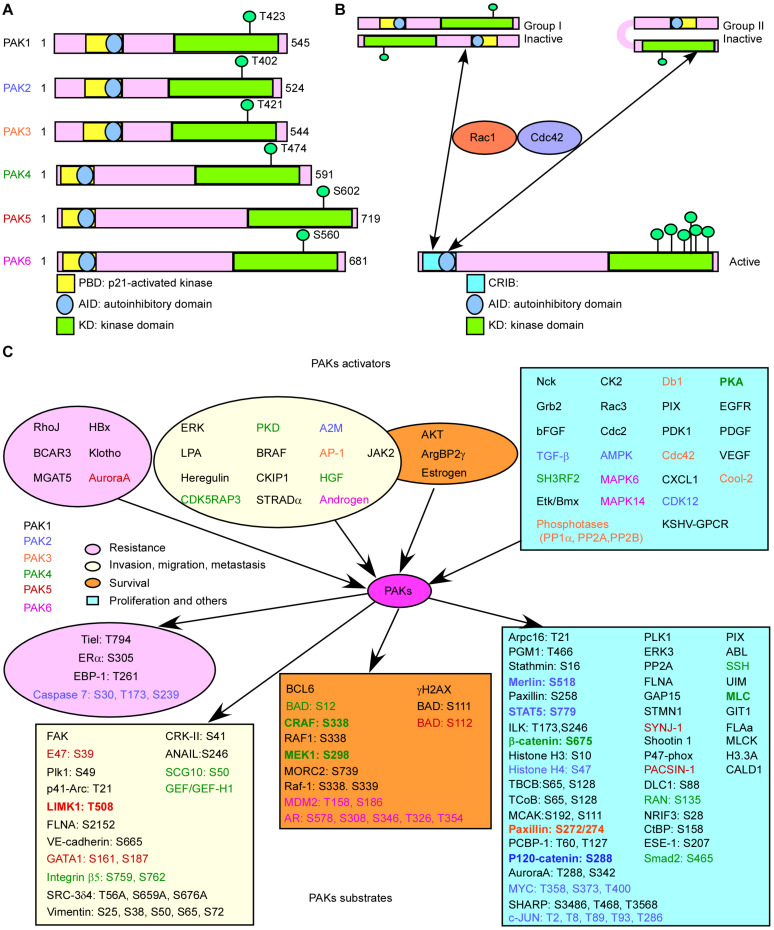
Basic characteristic of p21-activated kinases (PAKs). **(A)** The structures of PAKs. **(B)** The activation of PAKs. **(C)** The upstream activators and downstream effectors of PAKs with related functions.

All PAKs contain a carboxyl terminal kinase domain (KD), a p21-binding domain (PBD), and an auto inhibitory domain (AID) (Figure 1A; Radu et al., 2014). However, the regulatory domains of PAKs are structurally different, as well as the activation processes. The PBDs of group I PAKs overlap with the AIDs. Group I PAK activity is stimulated by binding to the activated small GTPase or some other proteins in the PBD, and concomitant interaction with the proximal amino acids and phosphoinositide, leading to dissociation of the AID from the KD. These changes will induce further conformational changes in the dimerized PAKs and cause activation of their kinase activity (Kumar et al., 2017). There are a lot of PAK activators, and downstream substrates have been identified. When bound to the substrate, the KD of PAKs becomes a monomer, and automatically phosphorylation stabilizes PAKs in the activated form (Kumar et al., 2017), while the overlapping of PBD and AID binds to KD of another PAK and forms inactive homo-dimerization (Figure 1B). PAK1 is activated by both GTPase-dependent and GTPase-independent manner via integrating multiple cell membrane receptor signals and proximal cytoplasmic signaling components (Kumar and Li, 2016). Due to the sequence similarities of group I PAKs, PAK1, PAK2, and PAK3 may share some mechanisms (King et al., 2014). The GTPase-independent activation of PAKs involves PXXP motifs in PAKs binding to Src homology 3 (SH3) adaptor proteins and phosphorylation of PAKs by other protein kinases, such as AKT, JAK2, PDK1, and ETK/BMX (Lu and Mayer, 1999; Zhou et al., 2003; Kumar and Li, 2016). Some other proteins stimulate group I PAK activity by protein–protein interaction at the SH3 domain, such as adapter proteins Grb2, Nck, and PIXs. Grb2 SH3 domain binds to the second proline-rich region of PAK1, stimulating EGFR (Puto et al., 2003). PAK1–Grb2 complex itself is independent of this stimulation, and Grb2 also mediates the interaction of activated EGFR and PAK1 (Puto et al., 2003). PAK1–Nck complex facilitates the cycling between cytosolic and focal complex sites (Zhao et al., 2000). PIXs (αPIX and βPIX), the Cdc42/Rac1 guanine nucleotide exchange factors (GEFs), bind with group I PAKs to regulate cell migration, immune system, and neuro system (Lucanic and Cheng, 2008; Santiago-Medina et al., 2013). Phosphorylation of PAK1 at T^402^ promotes the separation of PAK1 with Nck and PIXs, and phosphorylation of PAK1 at S^21^ by AKT inhibits the interaction between Nck and PAK1 (King et al., 2014). Group I PAK inactivation is controlled by protein phosphatases, such as PP2A, POPX1, POPX2, PKA, PP1α, and RhoV, as well as microRNA 7 and Merlin.

The structure of group II PAKs is different from that of group I PAKs, which contains an AID-like pseudosubstrate sequence that inactivates the Cdc42-bound PBD kinase activity. There is a lack of understanding on the activity controlling group II PAKs. A potential model proposed that group II PAKs function as a monomer rather than a dimer. The AID binds to KD in *cis*, which maintains the group II PAKs in an inactive form (Figure 1B; Baskaran et al., 2012). It is reported that PAK4 can be activated by HGF independent on PI3K, modulating cytoskeleton organization and cell adhesion (Wells et al., 2002). We need more evidences to uncover the group II PAK activity regulation due to the limited results.

## Function and Alterations of p21-Activated Kinases

Studies have shown that the activators and substrates of PAKs along with PAKs regulate mRNA splicing, gene expression, transcription, and translation and are involved in signal transduction, resulting in cell proliferation, cell growth, angiogenesis, metastasis, and drug resistance in cancer progression (Figure 1C), indicating that PAKs may play important roles in cancer (Supplementary Table 1; Furnari et al., 2014; Chetty et al., 2020; Quan et al., 2020). Knockout of PAK2 and PAK4 leads to embryonic lethality in transgenic mouse, indicating that they are critical for early development. Cell-specific genetic deletion of PAK2 in hematopoietic stem cells (HSCs) resulted in severe leukopenia and mild macro-cellular anemia via decreased expression of Jun-B and increased gene expression of c-Myc in a conditional PAK2 knockout mouse model (Zeng et al., 2015). Overexpression of different PAKs in different cancer types is co-related with cancer progression or drug resistance, and some of them can act as prognostic biomarkers for cancer (Supplementary Table 2).

**FIGURE 2:**
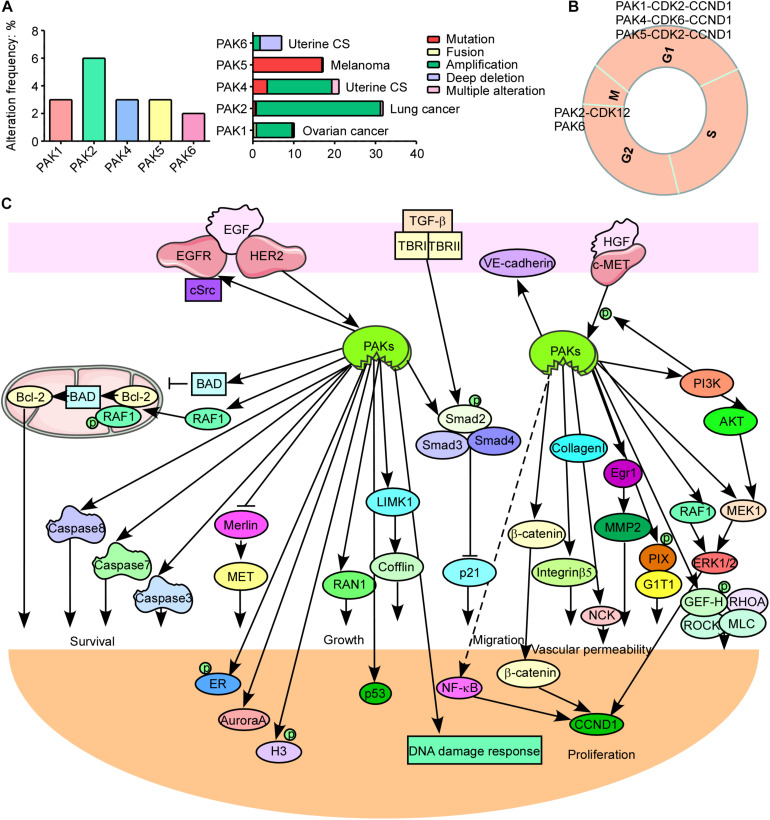
p21-activated kinases (PAKs) in cancer. **(A)** The genomic alterations of PAKs. **(B)** Correlation of PAKs with cyclin-dependent kinases (CDKs) and cyclins in cell cycle progression. **(C)** The PAK-mediated signaling pathways in cancer development.

PAK1, PAK2, and PAK4 have a higher expression level in most of cancer types, while other PAKs are not (Supplementary Figure 1). And PAK alterations including amplification, mutation, fusion, deep deletion, and multiple alterations frequently occur in cancers. PAK2 has the highest alteration frequency among all PAKs and most frequently in lung cancer (Figure 2A). PAK2 haploinsufficiency will lead to significant decreased synapse densities, defective long-term potentiation, and autism-related behaviors by regulating actin cytoskeleton dynamics in mice, indicating that PAK2 plays a key role in brain development and autism pathogenesis (Wang et al., 2018b). PAK1 mediates the phosphorylation of hypertrophy and/or arrhythmia-related proteins and plays important roles in cardiac diseases (Wang et al., 2018a). PAK3 mutations and PAK1 hyper-activation can cause MR such as X-linked MR (XLMR), Alzheimer disease (AD), and Huntington disease (HD) (Duarte et al., 2020; Zhang et al., 2020). Both PAK4 knockout and epiblast-specific deletion of PAK4 lead to embryonic lethality due to the abnormalities in the vasculature throughout the extraembryonic tissue and the epiblast (Tian et al., 2009). PAK2 and PAK4 were activated by gastrointestinal hormones/neurotransmitters and growth factors in pancreatic acinar diseases. And thus PAK2/PAK4 may be new therapeutic targets for the treatment of diseases involved in deregulation of pancreatic acinar cells (Nuche-Berenguer et al., 2016; Ramos-Alvarez and Jensen, 2018). PAK signaling pathway was reported to be involved in WNT and G-protein signaling in type 2 diabetes mellitus (T2DM) (Dammann et al., 2018). PAK5 was reported to be involved in T2DM, AD, psychosis, and cancer (Timm et al., 2006; Fadista et al., 2014; Morris et al., 2014). In contrast to the oncogenic function of other PAKs, PAK6 has both oncogenic and tumor suppressive functions in different cancers. It was reported that PAK6 promotes tumor progression and chemo-resistance in colon cancer and gastric cancer (GC), while decreased PAK6 expression was associated with tumor node metastasis stage progression and poor overall survival in clear cell renal cell carcinoma (ccRCC) and hepatocellular carcinoma (HCC) patients (Liu et al., 2014, 2015; Chen et al., 2015; Jiang et al., 2017). Besides, PAK6 controls neurite complexity in the healthy brain and leucine-rich repeat kinase 2 (LRRK2), a causative gene for Parkinson’s disease (PD) (Civiero et al., 2015). In addition, PAK1 and PAK2 have the maximum single-nucleotide polymorphisms (SNPs), while PAK3 has the minimum SNPs associated with human diseases among all PAKs according to genome-wide association study (GWAS) Atlas database.

## Molecular Mechanisms of p21-Activated Kinases’ Function in Cancer and Beyond

p21-activated kinases are the nexus of several pathogenic and oncogenic signaling pathways resulting in disease initiation and progression. Here, we review the molecular mechanisms of PAK’s function in different diseases including cancer, MR, cardiac disorders, diabetes, pancreatic acinar diseases, and viral infection. Better understanding of PAK-related pathological process will provide new insights into the therapy options as well as drug development for treating and preventing PAK-induced diseases.

### Cell Cycle and p21-Activated Kinases

The normal run of the cell cycles (G0/G1, S, G2, and M phases) ensures the normal proliferation of cells, while the abnormal regulation or expression of cell cycle-related proteins is a key hallmark for cancer. Cyclin-dependent kinases (CDKs), cyclins, checkpoint kinases, Aurora kinases, Polo-like kinases, and PAKs can directly or indirectly regulate the cell cycle processes. Interestingly, PAKs always combine with CDKs and cyclins to participate in cell cycle regulation (Figure 2B). PAK1 silencing leads to decreased cyclin E (CCNE) and CDK2, resulting in cell cycle arrest at the G1 and S phases in HCC (Zhang et al., 2018). It is reported that monocyte chemoattractant protein 1 (MCP1) activates PAK1 in a Rac1–NFATc1–CCND1–CDK6–CDK4-dependent manner in the mediation of human aortic smooth muscle cell migration and proliferation (Kundumani-Sridharan et al., 2013). PAK2 was reported to bind with CDK12 to activate MAPK signaling pathway, resulting in G2 phase dysregulation and GC progression (Liu et al., 2020). PAK4 upregulation contributes to cell cycle abnormality in the G0/G1 phase in oral squamous cell carcinoma (Peng and Pang, 2019). Knockdown of PAK5 induces cell cycle arrest at the G0/G1 phase, in concordance with the downregulation of CDK2, CDC25A, and cyclin D1 (CCND1) in GC (Gu et al., 2013). PAK6 knockdown causes cell cycle arrest at the G2/M phase in prostate cancer (Wen et al., 2009). PAK1 regulates mitosis via different proteins, including phosphorylating Aurora A at T^288^ and S^342^, binding and phosphorylating Tubulin cofactor B (TCoB) at S^65^ and S^128^, phosphorylating mitotic centromere-associated kinesin (MCAK) at S^192^ and S^111^, PLK1 activation, and microtubule dynamics regulation (Vadlamudi et al., 2005a; Zhao et al., 2005; Maroto et al., 2008; Pakala et al., 2012). In addition, PAK1 affects cell cycle via regulating chromosome dynamic changes, for example, as phosphorylation of histone 3 by PAK1 and phosphorylation of MORC2 at S^739^ (Li et al., 2002, 2012). Besides, PAK1 phosphorylates Raf at S^338^ to activate checkpoint kinase 2 (CHK2), leading to DNA damage response and cancer cell survival (Advani et al., 2015). These findings provide new thoughts that combining PAK inhibitor and cell cycle destruction may obtain dramatic benefits for cancer therapy.

### p21-Activated Kinases in Cancer

Cancer is one of the major health burdens worldwide. PAKs have been proved to be pivotal in cancer initiation, growth, angiogenesis, immunity, metabolism, metastasis, and drug resistance (Akkanapally et al., 2020; Bautista et al., 2020; Huang et al., 2020). PAKs may, through several signaling pathways, regulate cancer cell growth, apoptosis, or autophagy. These signal transductions mainly include WNT/β-catenin signaling pathway, EGFR/HER2/MAPK signaling pathway, PI3K/AKT signaling pathway, NF-κB cascades, and apoptosis, autophagy, epithelial–mesenchymal transition (EMT) signaling pathways, as well as DNA damage response signaling pathways (Figure 2C; Ishida et al., 2007; Won et al., 2019; Wu et al., 2019; Jiang et al., 2020; Li et al., 2020). PAK1 phosphorylates β-catenin at S^675^ and S^663^ to stabilize β-catenin and translocate to nuclear, activating transcription of targeted genes, containing c-MYC, CCND1, and MMP, leading to promoted cancer progression and drug resistance (He et al., 2008; Huynh et al., 2015, 2016; Semenova et al., 2017). In addition, PAK1 inhibits kinase activity of GSK3 by AKT1 activation, removing the inhibition effect on β-catenin (Manning and Toker, 2017). EGFR and HER2 are well-defined therapeutic targets for cancer. ERBB signaling pathway can activate and recruit various downstream proteins involved in different oncogenic signal transductions, such as MAPK and PI3K/AKT signaling pathways. PI3K phosphorylates some RhoGEFs that are able to activate Rac and then activate AKT and PAK1 (Fruman et al., 2017). PAK1 can activate Raf, MEK, and ERK; Raf in turn can activate PAK1 (El-Baba et al., 2014). The cross-talk between MAPK and PI3K/AKT signaling pathways contributes to cancer drug resistance, and the combination inhibition of PAKs and other proteins in the network may be a new strategy for cancer therapy to overcome drug resistance. PAKs can also stimulate JNK and NF-κB interacting kinase (NIK) to activate NF-κB signaling pathway, leading to cancer development (Dammann et al., 2014; Li et al., 2017). Thus, PAK1 inhibition might be a potential option for NF-κB-dependent cancer therapy. PAK1 phosphorylates Raf-1 at S^338^ and S^339^ to translocate it to mitochondria with phosphorylating BAD at S^112^, and Raf-1 in mitochondria disrupts Bcl-2–BAD complex formation to enhance the pro-survival ability of Bcl-2 (King et al., 2014). Besides, PAK1 phosphorylates BAD at S^111^, as well as DLC1, by interacting with Bim to resist apoptosis (Ong et al., 2011). PAKs also play important roles in autophagy directly or indirectly. PAK1 phosphorylates ATG5 at T^101^ to inhibit its ubiquitinated degradation and promote the core complexes (ATG5–ATG12–ATG16L) of autophagy with E3 ligase (Feng et al., 2020). Moreover, mTOR is a key autophagy-related biomarker, except for its role in cell growth regulator. It is reported that PAK1 inhibition leads to decreased mTOR activity and cytostatic autophagy, and regulation of PAK1 in other cascades (MAPK and NF-κB) can indirectly regulate autophagy (Dou et al., 2016; Wang et al., 2016, 2017). The loss of epithelial cell integrity resulting from degradation of the adhesive connections, which maintain contact between epithelial cells, is a hallmark of EMT, which is mainly accomplished by EMT promoting transcription factors (twist, snail, and slug) via PAK-activated signaling pathways, such as MAPK, PI3K/AKT, NF-κB, LIMK1/cofilin, and JNK signaling pathways (Yao et al., 2020). The cross-talk of PAKs with other signaling pathways is critical for cancer progression. PAK1 inhibits TGF-β expression and TGF-β in turn to enhance PAK1 activation to promote cancer development. It is also reported that a co-repressor of Notch signaling pathway was phosphorylated by PAK1 at S^3486^ and T^3568^ to inhibit Notch targeted gene activation and regulate cancer fate (Vadlamudi et al., 2005b). The cross-talk between PAK1 and STAT5 signal transductions is extremely important for leukemia, and the cross-talk between Hippo/YAP and FAK/ILK/PAK1 cascades promotes cell cytoskeleton modulation in liver cancer (Chatterjee et al., 2014; Sabra et al., 2017; Yuanxin et al., 2019). These findings suggest that targeting PAK-mediated signaling pathway along with other oncogenic cascades is a novel strategy for therapy of different cancers.

### p21-Activated Kinases in Infectious Diseases

Besides the critical roles in cancer, PAKs are important for infectious diseases through regulating several host-driven responses to pathogen infection, through anti-pathogen signal transduction and immune regulation (John Von Freyend et al., 2017). PAK1 and PAK2 affect infection progression (virus, bacteria, and parasitic protists) at various stages, promoting pathogens entry into, replication within, survival, and secretion from host cells. We summarize the major pathogens, such as hepatitis C virus (HCV), human hepatitis B virus (HBV), HIV, human papillomavirus (HPV), amphotropic murine leukemia virus (A-MLV), SARS, SARS-CoV-2, *Helicobacter pylori*, and *Leishmania*. We summarize related PAK signaling pathways in Supplementary Table 3 (Naranatt et al., 2005; Hoppe et al., 2006; Carter et al., 2011; Lee et al., 2013; Andrade et al., 2016; Sun et al., 2018). We use the example of *H. pylori* and viral (coronavirus) infection to discuss the role of PAKs in the infectious diseases.

*Helicobacter pylori* is a well-identified risk factor for gastritis and GC, which affects almost half of the population worldwide (Parsonnet et al., 1991). In epithelial cells, *H. pylori* activates Rac1 and stimulates PAK1 in a type 4 secretion system (T4SS)-dependent (Mejías-Luque et al., 2017) manner and causes PAK1–NIK interaction. Oncogenic protein CagA of *H. pylori* can interact with PAK1 to activate NF-κB, leading to motility, pro-inflammatory, and proliferative responses (Foryst-Ludwig and Naumann, 2000; Lim et al., 2009). In macrophage, purified lipopolysaccharide (LPS) from *H. pylori* stimulates Rac1, and PAK1 kinase activity triggers PAK1–caspase 1 interaction and phosphorylation at S^376^ and activates NF-κB to induce IL-1β secretion (Basak et al., 2005; Neumann et al., 2006; Supplementary Figure 2A). It was reported that PAK1 involved in the SARS-CoV-2 infection process and targeting PAK1 signaling pathway was verified to be effective for anti-SARS-CoV-2 treatment in preclinical trials (Berretta et al., 2020; Maruta and He, 2020). SARS-CoV-2 enters into host cells via binding to ACE2 and activated by TMPRSS2. Then PAK1 is activated to reduce the adaptive immune response against the virus. PAK1 can also stimulate CCL2 production, leading to a fibrotic response, and viral infection induces NF-κB activation to generate local pro-inflammatory cytokines production, such as IL-6 and IL-1β (Berretta et al., 2020; Supplementary Figure 2B). These findings suggest that targeting PAKs can be a novel therapy option for infectious diseases.

### p21-Activated Kinases in Mental Retardation, Cardiac Disorders, Diabetes, and Pancreatic Acinar Diseases

p21-activated kinases are also involved in other non-cancer diseases, which include MR, cardiac disorders, diabetes, and pancreatic acinar diseases. PAK dysregulation has been found in a variety of MR mainly including AD, PD, and HD. ROCK and PAK1 have been verified to be activated by fibrillar and β-amyloid (Aβ) oligomers, which in turn stimulate LIMK1 as well as induce cofilin phosphorylation, resulting in actin de-polymerization (Salminen et al., 2008). In addition, Aβ oligomer-induced effects on dendritic spine and synaptic marker missing are mediated by NMDA receptors via FYN–PAK interaction (Singh et al., 2017). Moreover, curcumin was reported to inhibit PAK1 activity through suppressing Aβ oligomer and fibril toxicity (Cai et al., 2009). These findings suggest that Rho-ROCK/LIMK/cofilin and RAC/PAK/LIMK/cofilin signal transduction dysregulation plays an important role in AD pathogenesis (Supplementary Figure 3A, left). PAK1 or PIX bind to wild-type and mutant-type HTT protein, which promotes HTT aggregation and increases HTT neuronal toxicity, leading to clinical behavioral, mental disorders and cognitive deficits in HD (Supplementary Figure 3A, right) (Luo et al., 2008). And PAK1 inhibition could suppress HTT aggregate accumulation as well as neuronal toxicity (Luo et al., 2008). Moreover, PAK1 is the binding partner of fragile X MR protein (FMR1) and the related protein FXR1. Transcriptional silencing of FMR1 is the most common genetic cause of fragile X syndrome (FXS), the most commonly inherited form of MR in humans (Say et al., 2010). One research group showed that in an FMR1 knockout FXS mouse model, a PAK1-dominant negative transgene rescues some of the behavioral abnormalities; this observation was confirmed by using a small molecule inhibitor that also has the same effect (Hayashi et al., 2007; Pyronneau et al., 2017). These results suggest that targeting PAKs is a promising strategy for treating and preventing mental diseases.

PAK1 plays a key role in cardiac diseases including pressure overload and adrenergic stress-induced hypertrophy and ischemia/reperfusion injury, as well as disrupted Ca^2+^ homoeostasis-related cardiac arrhythmias. PAK1 regulates the Ca^2+^ handling proteins by activating PP2A, resulting in phosphorylation of MLC2 and cTnT and de-phosphorylation of phosphor-lamban (RyR, LTCC, and cTnI) (Sheehan et al., 2009; DeSantiago et al., 2014, 2018). Besides, PAK1 activates SRF and regulates SERCA2a transcription via MKK4/7/JNK cascades to protect the heart from arrhythmias (Wang et al., 2014). Moreover, PAK1 negatively regulates calcineurin via Smad3/F-Bxo32 signal transduction to prevent hypertrophy (Chen et al., 2013; Tsui et al., 2015). And fingolimod [PAK1 activator, an Food and Drug Administration (FDA)-approved drug] was proved to prevent arrhythmias and cardiac hypertrophy (Yun et al., 2016; Chen et al., 2018; Supplementary Figure 3B). These results highlight the therapeutic potential of PAK1 signaling pathway in cardiac disease treatment and prevention.

Type 2 diabetes mellitus is a chronic inflammatory disease and a risk factor for cancer. It is considered that hyperglycemia improves cell cycle progression through PAK–β-catenin signaling pathway in early diabetes (Lv et al., 2018). Proliferation and inflammation driven by PAKs lead to oxidative stress and contribute to beta cell dysfunction and reduction of cell mass (Dammann et al., 2018). Moreover, nuclear β-catenin was identified to associate with FOXO transcription factors in oxidative stress, which modulates PAK expression (Essers et al., 2005). It is reported that PAK/p53/MDM2, PAK/RTK, and PAK/JNK signaling pathways participate in T2DM to reduce beta cell mass, induce apoptosis, and impede cell cycle progression (Schmidt et al., 2006; Schulthess et al., 2009; Mikawa et al., 2014; Supplementary Figure 3C). And this pathogenesis can be altered by pioglitazone (anti-diabetic drug) via reducing proliferation (Hussain et al., 2017). These findings indicate that PAK inhibitors may be useful for chemoprevention in patients with T2DM.

PAK2 and PAK4 have been gradually confirmed to be important for pancreatic acinar diseases among all PAKs. The CCK-stimulated PAK2 has been proved to be mediated by the activation of CDC42/Rac1, PKC, and Src in rat pancreatic acinar cells (Nuche-Berenguer and Jensen, 2015). This activation of PAK2 stimulates several signal cascades including MAPK, FAK, and PI3K–Akt pathways to mediate physiological or pathophysiological responses related to the onset of pancreatitis, which can be inhibited by a PAK inhibitor, suggesting that PAK2 can act as a new therapeutic target for the treatment of pancreatic acinar diseases (Nuche-Berenguer et al., 2016). Except the PAK2-mediated signaling pathways in pancreatic acinar cells, PAK4 can also be activated by stimulation of CREB, the VIP-/secretin-preferring receptors via EPAC, as well as regulate β-catenin signaling pathway to participate in Na^+^ and K^+^-ATPase activation, mediating the pancreatic fluid secretion (Supplementary Figure 3D; Ramos-Alvarez and Jensen, 2018; Ramos-Alvarez et al., 2019, 2020). These results together illustrate that PAKs play important roles in pancreatitis and pancreatic cancer growth, as well as enzyme secretion.

## Inhibitors of p21-Activated Kinases

Considering the critical roles of PAKs in multiple diseases that cover a large variety of population worldwide, mainly including cancer, infectious diseases, MR, cardiac disorders, diabetes, and pancreatic acinar diseases, drug discovery by targeting PAKs has made great progress. PAK inhibitors include ATP-competitive inhibitors, allosteric inhibitors, and natural blockers. The ATP-competitive PAK inhibitors can be divided into indolocarbazole-based inhibitors, aminopyrazole-based inhibitors, 2-amino pyrido[2,3-*d*]pyrimidine-7(8H)-one-based inhibitors, aminopyrimidine-based inhibitors, and other ATP-competitive inhibitors. ATP-competitive inhibitors and allosteric inhibitors are developed based on the structure of PAKs. Natural PAK blockers are compounds extracted from plants, which inhibit PAK activity or block PAK-mediated pathogenic signaling pathways (Ryu et al., 2014; Aboukameel et al., 2017; Li et al., 2020; Qin et al., 2020; Shahinozzaman et al., 2020). We summarize the PAK inhibitors, related mechanisms, and applicable diseases (Supplementary Table 4). Due to the distinct and overlapping functions of PAKs, the pan-PAK inhibitors may cause undesirable PK characteristics, consequent lack of tumor responses, and adverse side effects (mainly on cardiovascular and gastrointestinal function), leading to the termination of PF-3758309 clinical trial (Crawford et al., 2012; Rudolph et al., 2016; Rane and Minden, 2019). So far, just the KPT-9274, a PAK4 and NAMPT dual inhibitor, is ongoing in phase I clinical trials (NCT02702492 and NCT04281420) for solid tumors and non-Hodgkin’s lymphoma (NHL) (Abu Aboud et al., 2016; Li et al., 2019; Cordover et al., 2020). Recently, CP734, a small molecule, was found to target PAK1 ATPase activity by binding to V342 of PAK1 (Wang et al., 2020). Moreover, CP734 suppressed pancreatic tumor growth with little toxicity observed in mouse models and showed synergistic effects with gemcitabine or 5-fluorouracil on pancreatic cancer cells (Wang et al., 2020). Another study revealed that inhibition of AURKA and PAK1 synergistically decreased survival of cancer cells and tumor growth in luminal and HER2-enriched breast cancer patient-derived xenograft mouse models (Korobeynikov et al., 2019). It is also reported that combined inhibition of PAK1 and PARP had a synergistic effect on suppressing cancer cell proliferation and tumor growth in PAK1 overexpressed breast cancer (Villamar Cruz et al., 2016). Loss of PAK1 prolonged survival and showed loss of β-catenin in the MMTV-HER2 transgenic mice; when treated with small-molecule inhibitors of PAK or β-catenin, it showed tumor regression; and combined inhibition was synergistic in mice bearing xenografts of HER2-positive breast cancer cells (Arias-Romero et al., 2013). BCR-ABL1 tyrosine kinase inhibitor imatinib combined with PAK inhibitor IPA-3 increased the inhibition of growth and apoptosis of leukemia cells (Flis et al., 2019). In addition, combination of PAK inhibitors IPA-3 or OTSSP167 and PKCι inhibitor auranofin was highly synergistic in EGFR or KRAS mutant lung adenocarcinoma and squamous cell carcinoma cell lines and inhibited tumor growth in mouse models (Ito et al., 2019a,b). PAK1 inhibitor combined with chemotherapy gemcitabine synergistically inhibited cancer cell proliferation and tumor growth in pancreatic cancer (Yeo et al., 2016). These findings suggest that PAK inhibitors can be therapeutic and synergistic and overcome drug resistance agents for cancer therapy. In addition, five potential PAK1 inhibitors were found by using the water thermodynamic analysis (Biswal et al., 2020). Propolis is a well-studied natural extraction from bees, which contains different PAK1 blockers including CAPE, CA, ARC, triterpenes, and apigenin (Maruta, 2011). More importantly, CAPE, CA, and ARC show significant therapeutic effects on various diseases, such as cancers, infectious diseases (HIV, HPV, influenza virus, and so on), inflammatory diseases, and PAK1-dependent MR (AD, PD, and HD), as well as T2DM (Maruta, 2014). Most importantly, the spike protein of SARS-CoV-2 binds to ACE2 and is activated by TMPRSS2, and then several cascades are triggered, allowing viral endocytosis and PAK1 activation, leading to SARS-CoV-2 entry into host cells (Berretta et al., 2020). Propolis-derived compounds were proved to downregulate TMPRSS2 expression and the anchor ACE2, as well as suppress PAK1 activity, reduce pro-inflammatory cytokine overproduction, improve NF-κB and monocyte/macrophage immunomodulation, and promote the production of antibodies against SARS-CoV-2 (Berretta et al., 2020). As a result, a clinical trial (NCT04480593) of EPP-AF (Brazilian green propolis extract) was recently initiated in Brazil for the treatment of SARS-CoV-2 patients (Berretta et al., 2020; Maruta and He, 2020). Other PAK1 blockers identified that may be useful for SARS-CoV-2 epidemic include melatonin, ciclesonide, ivermectin, istodax (FK228), and hydroxychloroquine (HQ) (Maruta and He, 2020). We list the associated clinical trials of different PAKs in Supplementary Table 5 and the most relevant clinical trials around PAK regulators and effectors in Supplementary Table 6. These findings indicate that PAK inhibitors are promising drugs for a variety of human diseases and beyond with lack of verification.

## Discussion

Since the identification of PAK1 in 1994, other PAKs and PAK-related structure, function, and molecular mechanisms were discovered gradually. PAKs play critical roles in widespread human diseases including cancer, infectious diseases, inflammatory diseases, MR, cardiac disorders, diabetes, pancreatic acinar diseases, and some other diseases. PAK inhibitors are proved to be effective in PAK-related diseases. These scientific findings altogether elucidate that targeting PAKs is a promising therapeutic strategy for various diseases, and using PAK inhibitors is a new option for treating and preventing human diseases. However, there are still some problems in the process of comprehensive understanding and applying PAKs for human disease treatment. The function and detailed molecular mechanisms in biological and pathological processes of most PAKs remain deserted. Development of specific PAK inhibitors remains a significant challenge due to the high homology of different PAKs at the KD, which may cause pan-PAK inhibition and accompanying off-target effects and severe side effects. Various methods for drug discovery may be useful to overcome these problems. These methods include structure-based drug design (SBDD), fragment-based drug design (FBDD), virtual screening (VS), high-throughput screening (HTS), drug repurposing (DR), and proteolysis-targeting chimera (PROTAC). Among all these methods, PROTAC is a promising strategy for designing selective PAK inhibitors, which contain a small molecule to control intracellular PAKs through recruiting PAKs to the ubiquitin or proteasome system for selective degradation. However, to our knowledge, no PROTACs targeting PAKs have been reported. Targeted covalent inhibitors (TCIs) have been successfully developed as high-affinity and selective inhibitors of protein kinases, which typically act by undergoing an electrophilic addition with an active-site cysteine residue, and the electrophilic additions generally require deprotonation of the thiol to form a reactive anionic thiolate (Awoonor-Williams and Rowley, 2018). The design of a TCI begins with the identification of a druggable cysteine, so it may be a strategy to target reactive cysteines in PAKs to inhibit PAK activity. There are no reports about targeting *in vivo* PAKs with siRNA or CRISPR strategies to our knowledge. So there may be opportunities or developments in targeting *in vivo* PAKs with siRNA or CRISPR strategies for treatment of hematological, liver, and other diseases. Moreover, the cross-talk between PAKs and other oncogenic/pathogenic signaling pathways, such as CDKs/PAKs, EGFR/PAKs, and BRD4/PAKs, is very important in cancer progression and drug resistance. Combining PAK inhibitors with other specific small molecules may be a potential therapeutic option for cancer prevention and therapy. In addition, the role of PAKs in autophagy, immune escape, and infection process makes it possible that PAK inhibitors can be used for immunotherapy, to overcome developed drug resistance and infectious diseases. However, most of these inhibitors discovered via preclinical study still lack clinical trials. PAKs have become hallmark and therapeutic targets for a variety of diseases. Many inhibitors of PAKs can greatly enrich the development of new drugs for prevention and treatment of human diseases, such as cancer, infectious diseases, and other diseases.

## Author Contributions

HL wrote the manuscript. KL and ZD revised the manuscript. All authors contributed to the article and approved the submitted version.

## Conflict of Interest

The authors declare that the research was conducted in the absence of any commercial or financial relationships that could be construed as a potential conflict of interest.

## Supplementary Material

Supplementary Figure 1 Expression of PAKs in different cancer types. PAKs’ expression in different cancer tissues and normal tissues. ACC, adrenocortical carcinoma; BLCA, bladder urothelial carcinoma; BRCA, breast invasive carcinoma; CESC, cervical squamous cell carcinoma and endocervical adenocarcinoma; CHOL, cholangiocarcinoma; COAD, colon adenocarcinoma; DLBC, lymphoid neoplasm diffuse large B-cell lymphoma; ESCA, esophageal carcinoma; GBM, glioblastoma multiforme; HNSC, head and neck squamous cell carcinoma; KICH, kidney chromophobe; KIRC, kidney renal clear cell carcinoma; KIRP, kidney renal papillary cell carcinoma; LAML, acute myeloid leukemia; LGG, brain lower grade glioma; LIHC, liver hepatocellular carcinoma; LUAD, lung adenocarcinoma; LUSC, lung squamous cell carcinoma; OV, ovarian serous cystadenocarcinoma; PAAD, pancreatic adenocarcinoma; PCPG, pheochromocytoma and paraganglioma; PRAD, prostate adenocarcinoma; READ, rectum adenocarcinoma; SARC, sarcoma; SKCM, skin cutaneous melanoma; STAD, stomach adenocarcinoma; TGCT, testicular germ cell tumor; THCA, thyroid carcinoma; THYM, thymoma; UCEC, uterine corpus endometrial carcinoma; UCS, uterine carcinosarcoma.

Supplementary Figure 2 PAKs in infectious diseases. **(A)** PAK1 cascades in *H. pylori* infection induced pathogenesis progression. **(B)** PAK1 dependent signaling pathways in lung fibrosis.

Supplementary Figure 3 PAKs signaling pathways in diseases beyond cancer and infection. **(A)** PAKs signal transduction in AD (left) and HD (right) pathogenesis. **(B)** PAKs signaling pathways in heart diseases. **(C)** PAKs cascades in response to hyperglycemia. **(D)** PAKs signaling in pancreatic acinar diseases.

Supplementary Table 1 Basic characteristics of PAKs.

Supplementary Table 2 Expression and alteration of PAKs related to human cancers.

Supplementary Table 3 PAKs associated pathogens and related biological process as well as signaling pathways. HBV, human hepatitis B virus; HCV, hepatitis C virus; HHV-8, human herpes virus 8; HIV, human immunodeficiency virus; HSV, herpes simplex virus; A-MLV, Amphotropic murine leukemia virus; ASFV, African swine fever virus; VV, vaccinia virus; SGIV, Singapore grouper Iridovirus; HAdv-3, human adenovirus 3; EV1, echovirus 1; ZEBOV, Zaire ebola virus; EPEC, enteropathogenic *E. coli*; NTHi, non-typeable *H. influenza*.

Supplementary Table 4 Inhibitor of PAKs.

Supplementary Table 5 Clinical trials of PAKs.

Supplementary Table 6 Clinical trials of PAK regulators and effectors.

## References

[B1] Aboukameel A. Muqbil I. Senapedis W. Baloglu E. Landesman Y. Shacham S. (2017). Novel p21-Activated Kinase 4 (PAK4) allosteric modulators overcome drug resistance and stemness in pancreatic ductal adenocarcinoma. *Mol. Cancer Therapeut.* 16 76–87. 10.1158/1535-7163.mct-16-0205 28062705 PMC5221563

[B2] Abu Aboud O. Chen C. H. Senapedis W. Baloglu E. Argueta C. Weiss R. H. (2016). Dual and specific inhibition of NAMPT and PAK4 By KPT-9274 decreases kidney cancer growth. *Mol. Cancer Therapeut.* 15 2119–2129. 10.1158/1535-7163.mct-16-0197 27390344 PMC5010932

[B3] Advani S. J. Camargo M. F. Seguin L. Mielgo A. Anand S. Hicks A. M. (2015). Kinase-independent role for CRAF-driving tumour radioresistance via CHK2. *Nat. Commun.* 6:8154. 10.1038/ncomms9154 PMC4559870 26333361

[B4] Akkanapally V. B. A. Kanumuri R. Vuttaradhi V. K. D’Cruze L. Murugan S. (2020). Clinical evaluation of P21 Activated Kinase 1 (PAK1) activation in gliomas and its effect on cell proliferation. *Cancer Invest.* 39 98–113. 10.1080/07357907.2020.1858097 33251876

[B5] Andrade L. G. Albarnaz J. D. Mügge F. L. David B. A. Abrahão J. S. da Fonseca F. G. (2016). Vaccinia virus dissemination requires p21-activated kinase 1. *Arch. Virol.* 161 2991–3002. 10.1007/s00705-016-2996-3 27465567

[B6] Arias-Romero L. E. Villamar-Cruz O. Huang M. Hoeflich K. P. Chernoff J. (2013). Pak1 kinase links ErbB2 to β-catenin in transformation of breast epithelial cells. *Cancer Res.* 73 3671–3682. 10.1158/0008-5472.can-12-4453 23576562 PMC3687032

[B7] Awoonor-Williams E. Rowley C. N. (2018). How reactive are druggable cysteines in protein kinases? *J. Chem. Inform. Model.* 58 1935–1946. 10.1021/acs.jcim.8b00454 30118220

[B8] Basak C. Pathak S. K. Bhattacharyya A. Mandal D. Pathak S. Kundu M. (2005). NF-kappaB- and C/EBPbeta-driven interleukin-1beta gene expression and PAK1-mediated caspase-1 activation play essential roles in interleukin-1beta release from *Helicobacter pylori* lipopolysaccharide-stimulated macrophages. *J. Biol. Chem.* 280 4279–4288. 10.1074/jbc.m412820200 15561713

[B9] Baskaran Y. Ng Y. W. Selamat W. Ling F. T. Manser E. (2012). Group I and II mammalian PAKs have different modes of activation by Cdc42. *EMBO Rep.* 13 653–659. 10.1038/embor.2012.75 22653441 PMC3388789

[B10] Bautista L. Knippler C. M. Ringel M. D. (2020). p21-Activated kinases in thyroid cancer. *Endocrinology* 161:bqaa105. 10.1210/endocr/bqaa105 PMC7417880 32609833

[B11] Berretta A. A. Silveira M. A. D. Cóndor Capcha J. M. De Jong D. (2020). Propolis and its potential against SARS-CoV-2 infection mechanisms and COVID-19 disease: running title: propolis against SARS-CoV-2 infection and COVID-19. *Biomed. Pharmacother.* 131:110622. 10.1016/j.biopha.2020.110622 32890967 PMC7430291

[B12] Biswal J. Jayaprakash P. Suresh Kumar R. Venkatraman G. Poopandi S. Rangasamy R. (2020). Identification of Pak1 inhibitors using water thermodynamic analysis. *J. Biomol. Struct. Dyn.* 38 13–31. 10.1080/07391102.2019.1567393 30661460

[B13] Cai X. Z. Wang J. Li X. D. Wang G. L. Liu F. N. Cheng M. S. (2009). Curcumin suppresses proliferation and invasion in human gastric cancer cells by downregulation of PAK1 activity and cyclin D1 expression. *Cancer Biol. Ther.* 8 1360–1368. 10.4161/cbt.8.14.8720 19448398

[B14] Carter G. C. Bernstone L. Baskaran D. James W. (2011). HIV-1 infects macrophages by exploiting an endocytic route dependent on dynamin, Rac1 and Pak1. *Virology* 409 234–250. 10.1016/j.virol.2010.10.018 21056892

[B15] Chan P. M. Manser E. (2012). PAKs in human disease. *Prog. Mol. Biol. Transl. Sci.* 106 171–187. 10.1016/b978-0-12-396456-4.00011-0 22340718

[B16] Chatterjee A. Ghosh J. Ramdas B. Mali R. S. Martin H. Kobayashi M. (2014). Regulation of Stat5 by FAK and PAK1 in oncogenic FLT3- and KIT-Driven Leukemogenesis. *Cell Rep.* 9 1333–1348. 10.1016/j.celrep.2014.10.039 25456130 PMC4380442

[B17] Chen G. Chen X. Sukumar A. Gao B. Curley J. Schnaper H. W. (2013). TGFβ receptor I transactivation mediates stretch-induced Pak1 activation and CTGF upregulation in mesangial cells. *J. Cell Sci.* 126(Pt 16), 3697–3712. 10.1242/jcs.126714 23781022 PMC3744028

[B18] Chen J. Lu H. Yan D. Cui F. Wang X. Yu F. (2015). PAK6 increase chemoresistance and is a prognostic marker for stage II and III colon cancer patients undergoing 5-FU based chemotherapy. *Oncotarget* 6 355–367. 10.18632/oncotarget.2803 25426562 PMC4381600

[B19] Chen W. Ghobrial R. M. Li X. C. Kloc M. (2018). Inhibition of RhoA and mTORC2/Rictor by Fingolimod (FTY720) induces p21-activated kinase 1, PAK-1 and amplifies podosomes in mouse peritoneal macrophages. *Immunobiology* 223 634–647. 10.1016/j.imbio.2018.07.009 30005970

[B20] Chetty A. K. Sexton J. A. Ha B. H. Turk B. E. Boggon T. J. (2020). Recognition of physiological phosphorylation sites by p21-activated kinase 4. *J. Struct. Biol.* 211:107553. 10.1016/j.jsb.2020.107553 32585314 PMC7395882

[B21] Civiero L. Cirnaru M. D. Beilina A. Rodella U. Russo I. Belluzzi E. (2015). Leucine-rich repeat kinase 2 interacts with p21-activated kinase 6 to control neurite complexity in mammalian brain. *J. Neurochem.* 135 1242–1256. 10.1111/jnc.13369 26375402 PMC4715492

[B22] Cordover E. Wei J. Patel C. Shan N. L. Gionco J. Sargsyan D. (2020). KPT-9274, an inhibitor of PAK4 and NAMPT, leads to downregulation of mTORC2 in triple negative breast cancer cells. *Chem. Res. Toxicol.* 33 482–491. 10.1021/acs.chemrestox.9b00376 31876149 PMC9316853

[B23] Crawford J. J. Hoeflich K. P. Rudolph J. (2012). p21-Activated kinase inhibitors: a patent review. *Exp. Opin. Therapeut. Patents* 22 293–310. 10.1517/13543776.2012.668758 22404134

[B24] Dammann K. Khare V. Coleman C. Berdel H. Gasche C. (2018). p-21 activated kinase as a molecular target for chemoprevention in diabetes. *Geriatrics* 3:73. 10.3390/geriatrics3040073 31011108 PMC6371191

[B25] Dammann K. Khare V. Gasche C. (2014). Tracing PAKs from GI inflammation to cancer. *Gut* 63 1173–1184. 10.1136/gutjnl-2014-306768 24811999

[B26] DeSantiago J. Bare D. J. Varma D. Solaro R. J. Arora R. Banach K. (2018). Loss of p21-activated kinase 1 (Pak1) promotes atrial arrhythmic activity. *Heart Rhythm* 15 1233–1241. 10.1016/j.hrthm.2018.03.041 29625277 PMC7017889

[B27] DeSantiago J. Bare D. J. Xiao L. Ke Y. Solaro R. J. Banach K. (2014). p21-Activated kinase1 (Pak1) is a negative regulator of NADPH-oxidase 2 in ventricular myocytes. *J. Mol. Cell. Cardiol.* 67 77–85. 10.1016/j.yjmcc.2013.12.017 24380729 PMC3930036

[B28] Dou Q. Chen H. N. Wang K. Yuan K. Lei Y. Li K. (2016). Ivermectin induces cytostatic autophagy by blocking the PAK1/Akt axis in breast cancer. *Cancer Res.* 76 4457–4469. 10.1158/0008-5472.can-15-2887 27302166

[B29] Duarte K. Heide S. Poëa-Guyon S. Rousseau V. Depienne C. Rastetter A. (2020). PAK3 mutations responsible for severe intellectual disability and callosal agenesis inhibit cell migration. *Neurobiol. Dis.* 136:104709. 10.1016/j.nbd.2019.104709 31843706

[B30] El-Baba C. Mahadevan V. Fahlbusch F. B. Mohan S. S. Rau T. T. Gali-Muhtasib H. (2014). Thymoquinone-induced conformational changes of PAK1 interrupt prosurvival MEK-ERK signaling in colorectal cancer. *Mol. Cancer* 13:201. 10.1186/1476-4598-13-201 25174975 PMC4158125

[B31] Essers M. A. de Vries-Smits L. M. Barker N. Polderman P. E. Burgering B. M. Korswagen H. C. (2005). Functional interaction between beta-catenin and FOXO in oxidative stress signaling. *Science* 308 1181–1184. 10.1126/science.1109083 15905404

[B32] Fadista J. Vikman P. Laakso E. O. Mollet I. G. Esguerra J. L. Taneera J. (2014). Global genomic and transcriptomic analysis of human pancreatic islets reveals novel genes influencing glucose metabolism. *Proc. Natl. Acad. Sci. U.S.A.* 111 13924–13929. 10.1073/pnas.1402665111 25201977 PMC4183326

[B33] Feng X. Zhang H. Meng L. Song H. Zhou Q. Qu C. (2020). Hypoxia-induced acetylation of PAK1 enhances autophagy and promotes brain tumorigenesis via phosphorylating ATG5. *Autophagy* [Epub ahead of print]. 10.1080/15548627.2020.1731266 32186433 PMC8032228

[B34] Flis S. Bratek E. Chojnacki T. Piskorek M. Skorski T. (2019). Simultaneous inhibition of BCR-ABL1 tyrosine kinase and PAK1/2 serine/threonine kinase exerts synergistic effect against chronic myeloid leukemia cells. *Cancers* 11:1544. 10.3390/cancers11101544 31614827 PMC6826736

[B35] Foryst-Ludwig A. Naumann M. (2000). p21-activated kinase 1 activates the nuclear factor kappa B (NF-kappa B)-inducing kinase-Ikappa B kinases NF-kappa B pathway and proinflammatory cytokines in *Helicobacter* pylori infection. *J. Biol. Chem.* 275 39779–39785. 10.1074/jbc.m007617200 11016939

[B36] Fruman D. A. Chiu H. Hopkins B. D. Bagrodia S. Cantley L. C. Abraham R. T. (2017). The PI3K pathway in human disease. *Cell* 170 605–635. 10.1016/j.cell.2017.07.029 28802037 PMC5726441

[B37] Furnari M. A. Jobes M. L. Nekrasova T. Minden A. Wagner G. C. (2014). Differential sensitivity of Pak5, Pak6, and Pak5/Pak6 double-knockout mice to the stimulant effects of amphetamine and exercise-induced alterations in body weight. *Nutr. Neurosci.* 17 109–115. 10.1179/1476830513y.0000000072 23710594 PMC4365912

[B38] Geng N. Li Y. Zhang W. Wang F. Wang X. Jin Z. (2020). A PAK5-DNPEP-USP4 axis dictates breast cancer growth and metastasis. *Int. J. Cancer* 146 1139–1151. 10.1002/ijc.32523 31219614

[B39] Gu J. Li K. Li M. Wu X. Zhang L. Ding Q. (2013). A role for p21-activated kinase 7 in the development of gastric cancer. *FEBS J.* 280 46–55. 10.1111/febs.12048 23106939

[B40] Hayashi M. L. Rao B. S. Seo J. S. Choi H. S. Dolan B. M. Choi S. Y. (2007). Inhibition of p21-activated kinase rescues symptoms of fragile X syndrome in mice. *Proc. Natl. Acad. Sci. U.S.A.* 104 11489–11494. 10.1073/pnas.0705003104 17592139 PMC1899186

[B41] He H. Shulkes A. Baldwin G. S. (2008). PAK1 interacts with beta-catenin and is required for the regulation of the beta-catenin signalling pathway by gastrins. *Biochim. Biophys. Acta* 1783 1943–1954. 10.1016/j.bbamcr.2008.04.016 18515095

[B42] Hoppe S. Schelhaas M. Jaeger V. Liebig T. Petermann P. Knebel-Mörsdorf D. (2006). Early herpes simplex virus type 1 infection is dependent on regulated Rac1/Cdc42 signalling in epithelial MDCKII cells. *J. Gen. Virol.* 87(Pt 12), 3483–3494. 10.1099/vir.0.82231-0 17098962

[B43] Huang H. Xue Q. Du X. Cui J. Wang J. Cheng D. (2020). . p21-activated kinase 4 promotes the progression of esophageal squamous cell carcinoma by targeting LASP1. *Mol. Carcinogen.* 60 38–50. 10.1002/mc.23269 33289209 PMC7756368

[B44] Hussain M. Shad M. N. Akhtar L. (2017). Pioglitazone attenuates cardiometabolic risk factors in non-diabetic patients with dyslipidemia. *JPMA J. Pakistan Med. Assoc.* 67 1884–1888. 29256535

[B45] Huynh N. Beutler J. A. Shulkes A. Baldwin G. S. He H. (2015). Glaucarubinone inhibits colorectal cancer growth by suppression of hypoxia-inducible factor 1α and β-catenin via a p-21 activated kinase 1-dependent pathway. *Biochim. Biophys. Acta* 1853 157–165. 10.1016/j.bbamcr.2014.10.013 25409929 PMC7709143

[B46] Huynh N. Shulkes A. Baldwin G. He H. (2016). Up-regulation of stem cell markers by P21-activated kinase 1 contributes to 5-fluorouracil resistance of colorectal cancer. *Cancer Biol. Ther.* 17 813–823. 10.1080/15384047.2016.1195045 27260988 PMC5004687

[B47] Ishida H. Li K. Yi M. Lemon S. M. (2007). p21-activated kinase 1 is activated through the mammalian target of rapamycin/p70 S6 kinase pathway and regulates the replication of hepatitis C virus in human hepatoma cells. *J. Biol. Chem.* 282 11836–11848. 10.1074/jbc.m610106200 17255101

[B48] Ito M. Codony-Servat C. Codony-Servat J. Lligé D. Chaib I. Sun X. (2019a). Targeting PKCι-PAK1 signaling pathways in EGFR and KRAS mutant adenocarcinoma and lung squamous cell carcinoma. *Cell Commun. Signal. CCS* 17:137. 10.1186/s12964-019-0446-z PMC6819333 31660987

[B49] Ito M. Codony-Servat C. Karachaliou N. Rosell R. (2019b). Targeting PKCι-PAK1 in EGFR-mutation positive non-small cell lung cancer. *Transl. Lung Cancer Res.* 8 667–673. 10.21037/tlcr.2019.08.25 31737502 PMC6835115

[B50] Jiang S. Gao Y. Yu Q. H. Li M. Cheng X. Hu S. B. (2020). P-21-activated kinase 1 contributes to tumor angiogenesis upon photodynamic therapy via the HIF-1α/VEGF pathway. *Biochem. Biophys. Res. Commun.* 526 98–104. 10.1016/j.bbrc.2020.03.054 32197838

[B51] Jiang Y. Liu W. Li T. Hu Y. Chen S. Xi S. (2017). Prognostic and predictive value of p21-activated Kinase 6 associated support vector machine classifier in gastric cancer treated by 5-fluorouracil/oxaliplatin chemotherapy. *EBioMedicine* 22 78–88. 10.1016/j.ebiom.2017.06.028 28687498 PMC5552213

[B52] John Von Freyend S. Kwok-Schuelein T. Netter H. J. Haqshenas G. Semblat J. P. Doerig C. (2017). Subverting host cell P21-activated kinase: a case of convergent evolution across pathogens. *Pathogens* 6:17. 10.3390/pathogens6020017 28430160 PMC5488651

[B53] King H. Nicholas N. S. Wells C. M. (2014). Role of p-21-activated kinases in cancer progression. *Int. Rev. Cell Mol. Biol.* 309 347–387. 10.1016/b978-0-12-800255-1.00007-7 24529727

[B54] Korobeynikov V. Borakove M. Feng Y. Wuest W. M. Koval A. B. Nikonova A. S. (2019). Combined inhibition of Aurora A and p21-activated kinase 1 as a new treatment strategy in breast cancer. *Breast Cancer Res. Treat.* 177 369–382. 10.1007/s10549-019-05329-2 31254157 PMC6661032

[B55] Kumar R. Li D. Q. (2016). PAKs in human cancer progression: from inception to cancer therapeutic to future oncobiology. *Adv. Cancer Res.* 130 137–209. 10.1016/bs.acr.2016.01.002 27037753

[B56] Kumar R. Sanawar R. Li X. Li F. (2017). Structure, biochemistry, and biology of PAK kinases. *Gene* 605 20–31. 10.1016/j.gene.2016.12.014 28007610 PMC5250584

[B57] Kundumani-Sridharan V. Singh N. K. Kumar S. Gadepalli R. Rao G. N. (2013). Nuclear factor of activated T cells c1 mediates p21-activated kinase 1 activation in the modulation of chemokine-induced human aortic smooth muscle cell F-actin stress fiber formation, migration, and proliferation and injury-induced vascular wall remodeling. *J. Biol. Chem.* 288 22150–22162. 10.1074/jbc.m113.454082 23737530 PMC3724667

[B58] Lee J. H. Wittki S. Bräu T. Dreyer F. S. Krätzel K. Dindorf J. (2013). HIV Nef, paxillin, and Pak1/2 regulate activation and secretion of TACE/ADAM10 proteases. *Mol. Cell* 49 668–679. 10.1016/j.molcel.2012.12.004 23317503

[B59] Lee J. S. Mo Y. Gan H. Burgess R. J. Baker D. J. van Deursen J. M. (2019). Pak2 kinase promotes cellular senescence and organismal aging. *Proc. Natl. Acad. Sci. U.S.A.* 116 13311–13319. 10.1073/pnas.1903847116 31209047 PMC6613284

[B60] Li D. Q. Nair S. S. Ohshiro K. Kumar A. Nair V. S. Pakala S. B. (2012). MORC2 signaling integrates phosphorylation-dependent, ATPase-coupled chromatin remodeling during the DNA damage response. *Cell Rep.* 2 1657–1669. 10.1016/j.celrep.2012.11.018 23260667 PMC3554793

[B61] Li F. Adam L. Vadlamudi R. K. Zhou H. Sen S. Chernoff J. (2002). p21-activated kinase 1 interacts with and phosphorylates histone H3 in breast cancer cells. *EMBO Rep.* 3 767–773. 10.1093/embo-reports/kvf157 12151336 PMC1084211

[B62] Li L. H. Wu G. Y. Lu Y. Z. Chen X. H. Liu B. Y. Zheng M. H. (2017). p21-activated protein kinase 1 induces the invasion of gastric cancer cells through c-Jun NH2-terminal kinase-mediated activation of matrix metalloproteinase-2. *Oncol. Rep.* 38 193–200. 10.3892/or.2017.5643 28534988

[B63] Li N. Lopez M. A. Linares M. Kumar S. Oliva S. Martinez-Lopez J. (2019). Dual PAK4-NAMPT inhibition impacts growth and survival, and increases sensitivity to DNA-damaging agents in waldenström macroglobulinemia. *Clin. Cancer Res.* 25 369–377. 30206161 10.1158/1078-0432.CCR-18-1776 PMC6320280

[B64] Li R. Wang H. Wang J. Cheng M. (2020). PB-10, a thiazolo[4,5-d] pyrimidine derivative, targets p21-activated kinase 4 in human colorectal cancer cells. *Bioorg. Med. Chem. Lett.* 30:126807. 10.1016/j.bmcl.2019.126807 31740249

[B65] Li X. Minden A. (2003). Targeted disruption of the gene for the PAK5 kinase in mice. *Mol. Cell Biol.* 23 7134–7142. 10.1128/mcb.23.20.7134-7142.2003 14517284 PMC230317

[B66] Li Y. K. Zou J. Ye D. M. Zeng Y. Chen C. Y. Luo G. F. (2020). Human p21-activated kinase 5 (PAK5) expression and potential mechanisms in relevant cancers: basic and clinical perspectives for molecular cancer therapeutics. *Life Sci.* 241:117113. 10.1016/j.lfs.2019.117113 31805288

[B67] Lim J. W. Kim K. H. Kim H. (2009). alphaPix interacts with *Helicobacter pylori* CagA to induce IL-8 expression in gastric epithelial cells. *Scand. J. Gastroenterol.* 44 1166–1172. 10.1080/00365520903144398 19672789

[B68] Liu H. Shin S. H. Chen H. Liu T. Li Z. Hu Y. (2020). CDK12 and PAK2 as novel therapeutic targets for human gastric cancer. *Theranostics* 10 6201–6215. 10.7150/thno.46137 32483448 PMC7255043

[B69] Liu W. Liu H. Liu Y. Xu L. Zhang W. Zhu Y. (2014). Prognostic significance of p21-activated kinase 6 expression in patients with clear cell renal cell carcinoma. *Ann. Surg. Oncol.* 21(Suppl. 4), S575–S583. 24715215 10.1245/s10434-014-3680-z

[B70] Liu W. Liu Y. Liu H. Zhang W. Fu Q. Xu J. (2015). Tumor suppressive function of p21-activated Kinase 6 in hepatocellular carcinoma. *J. Biol. Chem.* 290 28489–28501. 10.1074/jbc.m115.658237 26442588 PMC4653705

[B71] Liu W. Zi M. Naumann R. Ulm S. Jin J. Taglieri D. M. (2011). Pak1 as a novel therapeutic target for antihypertrophic treatment in the heart. *Circulation* 124 2702–2715. 10.1161/circulationaha.111.048785 22082674 PMC3242076

[B72] Lu W. Mayer B. J. (1999). Mechanism of activation of Pak1 kinase by membrane localization. *Oncogene* 18 797–806. 10.1038/sj.onc.1202361 9989831

[B73] Lucanic M. Cheng H. J. (2008). A RAC/CDC-42-independent GIT/PIX/PAK signaling pathway mediates cell migration in *C. elegans*. *PLoS Genet.* 4:e1000269. 10.1371/journal.pgen.1000269 19023419 PMC2581894

[B74] Luo S. Mizuta H. Rubinsztein D. C. (2008). p21-activated kinase 1 promotes soluble mutant huntingtin self-interaction and enhances toxicity. *Hum. Mol. Genet.* 17 895–905. 10.1093/hmg/ddm362 18065495

[B75] Lv Z. Hu M. Fan M. Li X. Lin J. Zhen J. (2018). Podocyte-specific Rac1 deficiency ameliorates podocyte damage and proteinuria in STZ-induced diabetic nephropathy in mice. *Cell Death Dis.* 9:342. 10.1038/s41419-018-0353-z PMC5832796 29497040

[B76] Manning B. D. Toker A. (2017). AKT/PKB signaling: navigating the network. *Cell* 169 381–405. 10.1016/j.cell.2017.04.001 28431241 PMC5546324

[B77] Maroto B. Ye M. B. von Lohneysen K. Schnelzer A. Knaus U. G. (2008). P21-activated kinase is required for mitotic progression and regulates Plk1. *Oncogene* 27 4900–4908. 10.1038/onc.2008.131 18427546

[B78] Maruta H. (2011). Effective neurofibromatosis therapeutics blocking the oncogenic kinase PAK1. *Drug Discov. Therapeut.* 5 266–278. 10.5582/ddt.2011.v5.6.266 22466437

[B79] Maruta H. (2014). Herbal therapeutics that block the oncogenic kinase PAK1: a practical approach towards PAK1-dependent diseases and longevity. *Phytother. Res.* 28 656–672. 10.1002/ptr.5054 23943274

[B80] Maruta H. He H. (2020). PAK1-blockers: potential therapeutics against COVID-19. *Med. Drug Discov.* 6:100039. 10.1016/j.medidd.2020.100039 32313880 PMC7166201

[B81] Maruta H. Kittaka A. (2020). Chemical evolution for taming the ‘pathogenic kinase’ PAK1. *Drug Discov. Today* 25 959–964. 10.1016/j.drudis.2020.03.008 32348877 PMC7194552

[B82] Mejías-Luque R. Zöller J. Anderl F. Loew-Gil E. Vieth M. Adler T. (2017). Lymphotoxin β receptor signalling executes *Helicobacter pylori*-driven gastric inflammation in a T4SS-dependent manner. *Gut* 66 1369–1381. 10.1136/gutjnl-2015-310783 27196595

[B83] Meng J. Meng Y. Hanna A. Janus C. Jia Z. (2005). Abnormal long-lasting synaptic plasticity and cognition in mice lacking the mental retardation gene Pak3. *J. Neurosci.* 25 6641–6650. 10.1523/jneurosci.0028-05.2005 16014725 PMC6725420

[B84] Mikawa T. Maruyama T. Okamoto K. Nakagama H. Lleonart M. E. Tsusaka T. (2014). Senescence-inducing stress promotes proteolysis of phosphoglycerate mutase via ubiquitin ligase Mdm2. *J. Cell Biol.* 204 729–745. 10.1083/jcb.201306149 24567357 PMC3941061

[B85] Møller L. L. V. Jaurji M. Kjøbsted R. Joseph G. A. Madsen A. B. Knudsen J. R. (2020). Insulin-stimulated glucose uptake partly relies on p21-activated kinase (PAK)2, but not PAK1, in mouse skeletal muscle. *J. Physiol.* 598 5351–5377. 10.1113/jp280294 32844438 PMC7771197

[B86] Morrice N. A. Gabrielli B. Kemp B. E. Wettenhall R. E. A. (1994). cardiolipin-activated protein kinase from rat liver structurally distinct from the protein kinases C. *J. Biol. Chem.* 269 20040–20046. 10.1016/s0021-9258(17)32124-5 8051089

[B87] Morris D. W. Pearson R. D. Cormican P. Kenny E. M. O’Dushlaine C. T. Perreault L. P. (2014). An inherited duplication at the gene p21 Protein-Activated Kinase 7 (PAK7) is a risk factor for psychosis. *Hum. Mol. Genet.* 23 3316–3326. 24474471 10.1093/hmg/ddu025 PMC4030770

[B88] Naranatt P. P. Krishnan H. H. Smith M. S. Chandran B. (2005). Kaposi’s sarcoma-associated herpesvirus modulates microtubule dynamics via RhoA-GTP-diaphanous 2 signaling and utilizes the dynein motors to deliver its DNA to the nucleus. *J. Virol.* 79 1191–1206. 10.1128/jvi.79.2.1191-1206.2005 15613346 PMC538527

[B89] Neumann M. Foryst-Ludwig A. Klar S. Schweitzer K. Naumann M. (2006). The PAK1 autoregulatory domain is required for interaction with NIK in *Helicobacter pylori*-induced NF-kappaB activation. *Biol. Chem.* 387 79–86. 16497167 10.1515/BC.2006.011

[B90] Nuche-Berenguer B. Jensen R. T. (2015). Gastrointestinal hormones/neurotransmitters and growth factors can activate P21 activated kinase 2 in pancreatic acinar cells by novel mechanisms. *Biochim. Biophys. Acta* 1853(10 Pt A), 2371–2382. 10.1016/j.bbamcr.2015.05.011 25979836 PMC5474308

[B91] Nuche-Berenguer B. Ramos-Álvarez I. Jensen R. T. (2016). The p21-activated kinase, PAK2, is important in the activation of numerous pancreatic acinar cell signaling cascades and in the onset of early pancreatitis events. *Biochim. Biophys. Acta* 1862 1122–1136. 10.1016/j.bbadis.2016.02.008 26912410 PMC4846574

[B92] Ong C. C. Jubb A. M. Haverty P. M. Zhou W. Tran V. Truong T. (2011). Targeting p21-activated kinase 1 (PAK1) to induce apoptosis of tumor cells. *Proc. Natl. Acad. Sci. U.S.A.* 108 7177–7182. 10.1073/pnas.1103350108 21482786 PMC3084065

[B93] Pakala S. B. Nair V. S. Reddy S. D. Kumar R. (2012). Signaling-dependent phosphorylation of mitotic centromere-associated kinesin regulates microtubule depolymerization and its centrosomal localization. *J. Biol. Chem.* 287 40560–40569. 10.1074/jbc.m112.399576 23055517 PMC3504770

[B94] Parsonnet J. Friedman G. D. Vandersteen D. P. Chang Y. Vogelman J. H. Orentreich N. (1991). *Helicobacter* pylori infection and the risk of gastric carcinoma. *N. Engl. J. Med.* 325 1127–1131. 1891020 10.1056/NEJM199110173251603

[B95] Peng M. Pang C. (2019). MicroRNA-140-5p inhibits the tumorigenesis of oral squamous cell carcinoma by targeting p21-activated kinase 4. *Cell Biol. Int.* [Epub ahead of print]. 10.1002/cbin.11213 31393040

[B96] Puto L. A. Pestonjamasp K. King C. C. Bokoch G. M. (2003). p21-activated kinase 1 (PAK1) interacts with the Grb2 adapter protein to couple to growth factor signaling. *J. Biol. Chem.* 278 9388–9393. 10.1074/jbc.m208414200 12522133

[B97] Pyronneau A. He Q. Hwang J. Y. Porch M. Contractor A. Zukin R. S. (2017). Aberrant Rac1-cofilin signaling mediates defects in dendritic spines, synaptic function, and sensory perception in fragile X syndrome. *Sci. Signal.* 10:eaan0852. 10.1126/scisignal.aan0852 29114038 PMC5988355

[B98] Qin Q. Wu T. Yin W. Sun Y. Zhang X. Wang R. (2020). Discovery of 2,4-diaminopyrimidine derivatives targeting p21-activated kinase 4: biological evaluation and docking studies. *Archiv. der Pharm.* 353:e2000097. 10.1002/ardp.202000097 32627873

[B99] Quan L. Cheng Z. Dai Y. Jiao Y. Shi J. Fu L. (2020). Prognostic significance of PAK family kinases in acute myeloid leukemia. *Cancer Gene Ther.* 27 30–37. 10.1038/s41417-019-0090-1 30890765

[B100] Radu M. Semenova G. Kosoff R. Chernoff J. (2014). Signalling during the development and progression of cancer. *Nat. Rev. Cancer* 14 13–25. 10.1038/nrc3645 24505617 PMC4115244

[B101] Ramos-Alvarez I. Jensen R. T. (2018). P21-activated kinase 4 in pancreatic acinar cells is activated by numerous gastrointestinal hormones/neurotransmitters and growth factors by novel signaling, and its activation stimulates secretory/growth cascades. *Am. J. Physiol. Gastrointest. Liver Physiol.* 315 G302–G317. 29672153 10.1152/ajpgi.00005.2018 PMC6139648

[B102] Ramos-Alvarez I. Lee L. Jensen R. T. (2019). Cyclic AMP-dependent protein kinase A and EPAC mediate VIP and secretin stimulation of PAK4 and activation of Na(+),K(+)-ATPase in pancreatic acinar cells. *Am. J. Physiol. Gastrointest. Liver Physiol.* 316 G263–G277. 30520694 10.1152/ajpgi.00275.2018 PMC6397337

[B103] Ramos-Álvarez I. Lee L. Jensen R. T. (2020). Group II p21-activated kinase, PAK4, is needed for activation of focal adhesion kinases, MAPK, GSK3, and β-catenin in rat pancreatic acinar cells. *Am. J. Physiol. Gastrointest. Liver Physiol.* 318 G490–G503. 31984786 10.1152/ajpgi.00229.2019 PMC7099487

[B104] Rane C. K. Minden A. (2019). P21 activated kinase signaling in cancer. *Semin. Cancer Biol.* 54 40–49. 10.1016/j.semcancer.2018.01.006 29330094

[B105] Ruan P. Dai X. Sun J. He C. Huang C. Zhou R. (2020). Integration of hepatitis B virus DNA into p21-activated kinase 3 (PAK3) gene in HepG2.2.15 cells. *Virus Genes* 56 168–173. 10.1007/s11262-019-01725-4 31897927

[B106] Rudolph J. Murray L. J. Ndubaku C. O. O’Brien T. Blackwood E. Wang W. (2016). Chemically diverse group I p21-Activated Kinase (PAK) inhibitors impart acute cardiovascular toxicity with a narrow therapeutic window. *J. Med. Chem.* 59 5520–5541. 10.1021/acs.jmedchem.6b00638 27167326

[B107] Ryu B. J. Kim S. Min B. Kim K. Y. Lee J. S. Park W. J. (2014). Discovery and the structural basis of a novel p21-activated kinase 4 inhibitor. *Cancer Lett.* 349 45–50. 10.1016/j.canlet.2014.03.024 24704155

[B108] Sabra H. Brunner M. Mandati V. Wehrle-Haller B. Lallemand D. Ribba A. S. (2017). β1 integrin-dependent Rac/group I PAK signaling mediates YAP activation of Yes-associated protein 1 (YAP1) via NF2/merlin. *J. Biol. Chem.* 292 19179–19197. 10.1074/jbc.m117.808063 28972170 PMC5702661

[B109] Salminen A. Suuronen T. Kaarniranta K. (2008). ROCK, PAK, and Toll of synapses in Alzheimer’s disease. *Biochem. Biophys. Res. Commun.* 371 587–590. 10.1016/j.bbrc.2008.04.148 18466762

[B110] Santiago-Medina M. Gregus K. A. Gomez T. M. (2013). Interactions regulate adhesion dynamics and membrane protrusion to control neurite outgrowth. *J. Cell Sci.* 126(Pt 5), 1122–1133. 10.1242/jcs.112607 23321640 PMC3635460

[B111] Say E. Tay H. G. Zhao Z. S. Baskaran Y. Li R. Lim L. (2010). A functional requirement for PAK1 binding to the KH(2) domain of the fragile X protein-related FXR1. *Mol. Cell* 38 236–249. 10.1016/j.molcel.2010.04.004 20417602

[B112] Schmidt M. H. H. Husnjak K. Szymkiewicz I. Haglund K. Dikic I. (2006). Cbl escapes Cdc42-mediated inhibition by downregulation of the adaptor molecule betaPix. *Oncogene* 25 3071–3078. 10.1038/sj.onc.1209329 16407834

[B113] Schulthess F. T. Paroni F. Sauter N. S. Shu L. Ribaux P. Haataja L. (2009). CXCL10 impairs beta cell function and viability in diabetes through TLR4 signaling. *Cell Metab.* 9 125–139. 10.1016/j.cmet.2009.01.003 19187771

[B114] Semenova G. Stepanova D. S. Dubyk C. Handorf E. Deyev S. M. Lazar A. J. (2017). Targeting group I p21-activated kinases to control malignant peripheral nerve sheath tumor growth and metastasis. *Oncogene* 36 5421–5431. 10.1038/onc.2017.143 28534510 PMC5608634

[B115] Shahinozzaman M. Ishii T. Ahmed S. Halim M. A. Tawata S. A. (2020). computational approach to explore and identify potential herbal inhibitors for the p21-activated kinase 1 (PAK1). *J. Biomol. Struct. Dyn.* 38 3514–3526. 10.1080/07391102.2019.1659855 31448698

[B116] Sheehan K. A. Ke Y. Wolska B. M. Solaro R. J. (2009). Expression of active p21-activated kinase-1 induces Ca2+ flux modification with altered regulatory protein phosphorylation in cardiac myocytes. *Am. J. Phys. Cell Physiol.* 296 C47–C58. 10.1152/ajpcell.00012.2008 PMC2636994 18923061

[B117] Singh N. K. Janjanam J. Rao G. N. (2017). p115 RhoGEF activates the Rac1 GTPase signaling cascade in MCP1 chemokine-induced vascular smooth muscle cell migration and proliferation. *J. Biol. Chem.* 292 14080–14091. 10.1074/jbc.m117.777896 28655771 PMC5572933

[B118] Sun H. Kamanova J. Lara-Tejero M. Galán J. E. (2018). *Salmonella* stimulates pro-inflammatory signalling through p21-activated kinases bypassing innate immune receptors. *Nat. Microbiol.* 3 1122–1130. 10.1038/s41564-018-0246-z 30224799 PMC6158040

[B119] Tian Y. Lei L. Cammarano M. Nekrasova T. Minden A. (2009). Essential role for the Pak4 protein kinase in extraembryonic tissue development and vessel formation. *Mech. Dev.* 126 710–720. 10.1016/j.mod.2009.05.002 19464366

[B120] Timm T. Matenia D. Li X. Y. Griesshaber B. Mandelkow E. M. (2006). Signaling from MARK to tau: regulation, cytoskeletal crosstalk, and pathological phosphorylation. *Neuro Degen. Dis.* 3 207–217. 10.1159/000095258 17047359

[B121] Tsui H. Zi M. Wang S. Chowdhury S. K. Prehar S. Liang Q. (2015). Smad3 couples Pak1 with the antihypertrophic pathway through the E3 ubiquitin ligase, Fbxo32. *Hypertension* 66 1176–1183. 10.1161/hypertensionaha.115.06068 26483344

[B122] Vadlamudi R. K. Barnes C. J. Rayala S. Li F. Balasenthil S. Marcus S. (2005a). p21-activated kinase 1 regulates microtubule dynamics by phosphorylating tubulin cofactor B. *Mol. Cell Biol.* 25 3726–3736. 10.1128/mcb.25.9.3726-3736.2005 15831477 PMC1084301

[B123] Vadlamudi R. K. Manavathi B. Singh R. R. Nguyen D. Li F. Kumar R. (2005b). An essential role of Pak1 phosphorylation of SHARP in Notch signaling. *Oncogene* 24 4591–4596. 10.1038/sj.onc.1208672 15824732

[B124] Villamar Cruz O. Prudnikova T. Y. Araiza-Olivera D. Perez-Plasencia C. Johnson N. Bernhardy A. J. (2016). Reduced PAK1 activity sensitizes FA/BRCA-proficient breast cancer cells to PARP inhibition. *Oncotarget* 7 76590–76603. 10.18632/oncotarget.12576 27740936 PMC5363532

[B125] Wang J. Zhu Y. Chen J. Yang Y. Zhu L. Zhao J. (2020). Identification of a novel PAK1 inhibitor to treat pancreatic cancer. *Acta Pharmaceut. Sin. B* 10 603–614. 10.1016/j.apsb.2019.11.015 32322465 PMC7161699

[B126] Wang K. Gao W. Dou Q. Chen H. Li Q. Nice E. C. (2016). Ivermectin induces PAK1-mediated cytostatic autophagy in breast cancer. *Autophagy* 12 2498–2499. 10.1080/15548627.2016.1231494 27657889 PMC5173258

[B127] Wang Y. Tsui H. Ke Y. Shi Y. Li Y. Davies L. (2014). Pak1 is required to maintain ventricular Ca^2 +^ homeostasis and electrophysiological stability through SERCA2a regulation in mice. *Circ. Arrhyth. Electrophysiol.* 7 938–948. 10.1161/CIRCEP.113.001198 PMC4213946 25217043

[B128] Wang Y. Wang S. Lei M. Boyett M. Tsui H. Liu W. (2018a). The p21-activated kinase 1 (Pak1) signalling pathway in cardiac disease: from mechanistic study to therapeutic exploration. *Br. J. Pharmacol.* 175 1362–1374. 10.1111/bph.13872 28574147 PMC5867015

[B129] Wang Y. Zeng C. Li J. Zhou Z. Ju X. Xia S. (2018b). PAK2 haploinsufficiency results in synaptic cytoskeleton impairment and autism-related behavior. *Cell Rep.* 24 2029–2041. 10.1016/j.celrep.2018.07.061 30134165

[B130] Wang Z. Jia G. Li Y. Liu J. Luo J. Zhang J. (2017). Clinicopathological signature of p21-activated kinase 1 in prostate cancer and its regulation of proliferation and autophagy via the mTOR signaling pathway. *Oncotarget* 8 22563–22580. 10.18632/oncotarget.15124 28186966 PMC5410245

[B131] Wells C. M. Abo A. Ridley A. J. (2002). PAK4 is activated via PI3K in HGF-stimulated epithelial cells. *J. Cell Sci.* 115(Pt 20), 3947–3956. 10.1242/jcs.00080 12244132

[B132] Wen X. Li X. Liao B. Liu Y. Wu J. Yuan X. (2009). Knockdown of p21-activated kinase 6 inhibits prostate cancer growth and enhances chemosensitivity to docetaxel. *Urology* 73 1407–1411. 10.1016/j.urology.2008.09.041 19362342

[B133] Won S. Y. Park J. J. Shin E. Y. Kim E. G. (2019). PAK4 signaling in health and disease: defining the PAK4-CREB axis. *Exp. Mol. Med.* 51 1–9. 10.1038/s12276-018-0204-0 30755582 PMC6372590

[B134] Wu H. Y. Yang M. C. Ding L. Y. Chen C. S. Chu P. C. (2019). p21-Activated kinase 3 promotes cancer stem cell phenotypes through activating the Akt-GSK3β-β-catenin signaling pathway in pancreatic cancer cells. *Cancer Lett.* 456 13–22. 10.1016/j.canlet.2019.04.026 31051214

[B135] Yao D. Li C. Rajoka M. S. R. He Z. Huang J. Wang J. (2020). P21-Activated Kinase 1: emerging biological functions and potential therapeutic targets in Cancer. *Theranostics* 10 9741–9766. 10.7150/thno.46913 32863957 PMC7449905

[B136] Yeo D. He H. Patel O. Lowy A. M. Baldwin G. S. Nikfarjam M. (2016). FRAX597, a PAK1 inhibitor, synergistically reduces pancreatic cancer growth when combined with gemcitabine. *BMC Cancer* 16:24. 10.1186/s12885-016-2057-z 26774265 PMC4715347

[B137] Yuanxin Y. Yanhong Z. Qin Z. Sishi T. Yang D. Yi Z. (2019). Pak1 gene functioned differentially in different BCR-ABL subtypes in leukemiagenesis and treatment response through STAT5 pathway. *Leukemia Res.* 79 6–16. 10.1016/j.leukres.2019.01.012 30784762

[B138] Yun J. Kim S. Y. Yoon K. S. Shin H. Jeong H. S. Chung H. (2016). P21 (Cdc42/Rac)-activated kinase 1 (pak1) is associated with cardiotoxicity induced by antihistamines. *Arch. Pharm. Res.* 39 1644–1652. 10.1007/s12272-016-0840-7 27681411

[B139] Zeng Y. Broxmeyer H. E. Staser K. Chitteti B. R. Park S. J. Hahn S. (2015). Pak2 regulates hematopoietic progenitor cell proliferation, survival, and differentiation. *Stem Cells* 33 1630–1641. 10.1002/stem.1951 25586960 PMC4409559

[B140] Zhang K. Wang Y. Fan T. Zeng C. Sun Z. S. (2020). The p21-activated kinases in neural cytoskeletal remodeling and related neurological disorders. *Protein Cell* [Epub ahead of print]. 10.1007/s13238-020-00812-9. 33306168 PMC8776968

[B141] Zhang Z. L. Liu G. C. Peng L. Zhang C. Jia Y. M. Yang W. H. (2018). Effect of PAK1 gene silencing on proliferation and apoptosis in hepatocellular carcinoma cell lines MHCC97-H and HepG2 and cells in xenograft tumor. *Gene Ther.* 25 284–296. 10.1038/s41434-018-0016-9 29802374

[B142] Zhao Z. S. Lim J. P. Ng Y. W. Lim L. Manser E. (2005). The GIT-associated kinase PAK targets to the centrosome and regulates Aurora-A. *Mol. Cell* 20 237–249. 10.1016/j.molcel.2005.08.035 16246726

[B143] Zhao Z. S. Manser E. Lim L. (2000). Interaction between PAK and nck: a template for Nck targets and role of PAK autophosphorylation. *Mol. Cell Biol.* 20 3906–3917. 10.1128/mcb.20.11.3906-3917.2000 10805734 PMC85736

[B144] Zheng J. Zhang C. Li Y. Jiang Y. Xing B. Du X. (2020). p21-activated kinase 6 controls mitosis and hepatocellular carcinoma progression by regulating Eg5. *Biochim. Biophys. Acta Mol. Cell Res.* 1868:118888. 10.1016/j.bbamcr.2020.118888 33098954

[B145] Zhou G. L. Zhuo Y. King C. C. Fryer B. H. Bokoch G. M. Field J. (2003). Akt phosphorylation of serine 21 on Pak1 modulates Nck binding and cell migration. *Mol. Cell Biol.* 23 8058–8069. 10.1128/mcb.23.22.8058-8069.2003 14585966 PMC262366

