# Supplementary figures and images for: The Role of p21-Activated Kinases in Cancer and Beyond: Where Are We Heading?
Source: Front Cell Dev Biol. 2021 Mar 16;9:641381. doi: 10.3389/fcell.2021.641381 (PMC8007885; doi:10.3389/fcell.2021.641381)

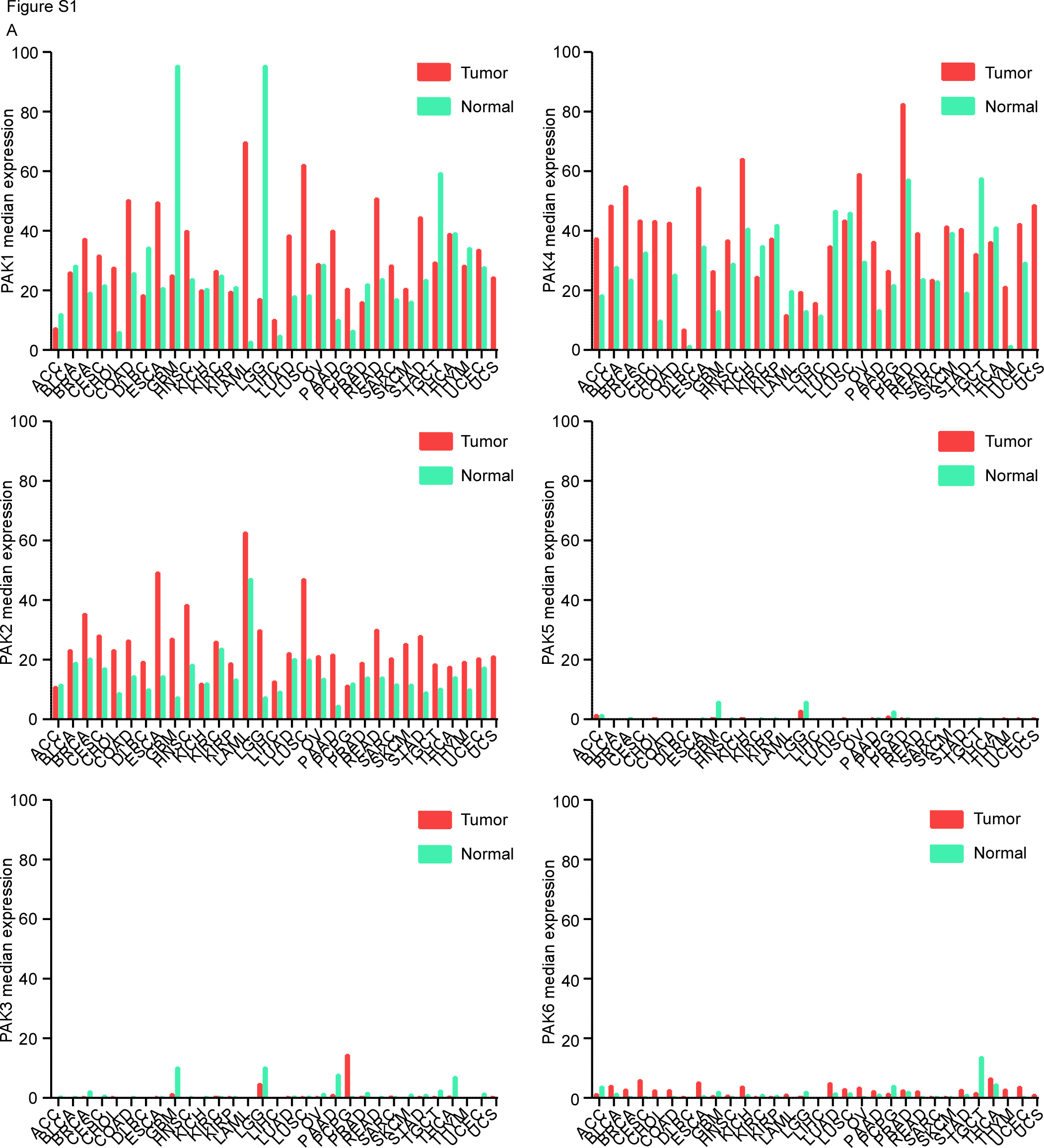

Supplement: Supplementary Figure 1 — Expression of PAKs in different cancer types. PAKs’ expression in different cancer tissues and normal tissues. ACC, adrenocortical carcinoma; BLCA, bladder urothelial carcinoma; BRCA, breast invasive carcinoma; CESC, cervical squamous cell carcinoma and endocervical adenocarcinoma; CHOL, cholangiocarcinoma; COAD, colon adenocarcinoma; DLBC, lymphoid neoplasm diffuse large B-cell lymphoma; ESCA, esophageal carcinoma; GBM, glioblastoma multiforme; HNSC, head and neck squamous cell carcinoma; KICH, kidney chromophobe; KIRC, kidney renal clear cell carcinoma; KIRP, kidney renal papillary cell carcinoma; LAML, acute myeloid leukemia; LGG, brain lower grade glioma; LIHC, liver hepatocellular carcinoma; LUAD, lung adenocarcinoma; LUSC, lung squamous cell carcinoma; OV, ovarian serous cystadenocarcinoma; PAAD, pancreatic adenocarcinoma; PCPG, pheochromocytoma and paraganglioma; PRAD, prostate adenocarcinoma; READ, rectum adenocarcinoma; SARC, sarcoma; SKCM, skin cutaneous melanoma; STAD, stomach adenocarcinoma; TGCT, testicular germ cell tumor; THCA, thyroid carcinoma; THYM, thymoma; UCEC, uterine corpus endometrial carcinoma; UCS, uterine carcinosarcoma. [file Image_1.TIF]

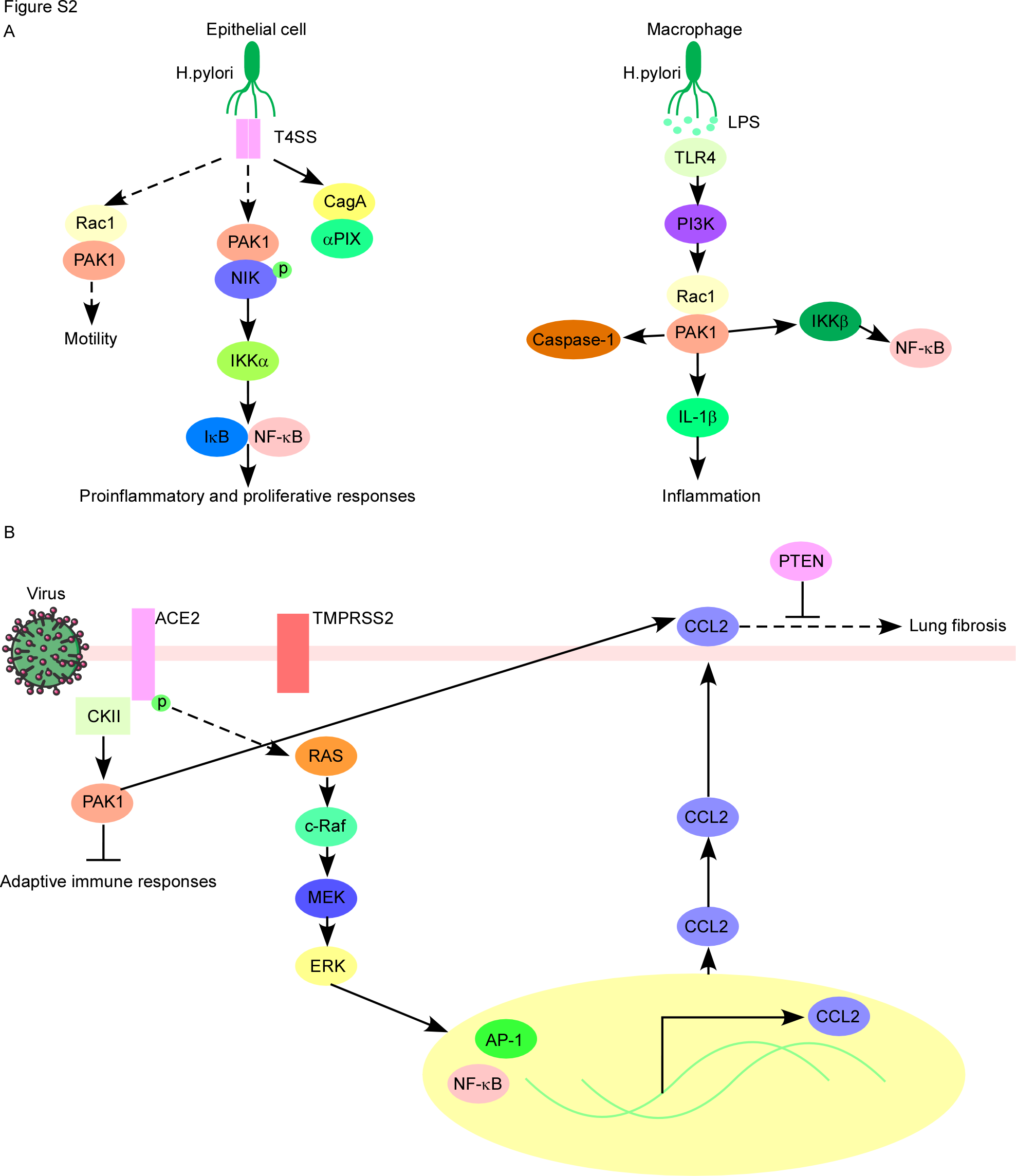

Supplement: Supplementary Figure 2 — PAKs in infectious diseases. (A) PAK1 cascades in H. pylori infection induced pathogenesis progression. (B) PAK1 dependent signaling pathways in lung fibrosis. [file Image_2.TIF]

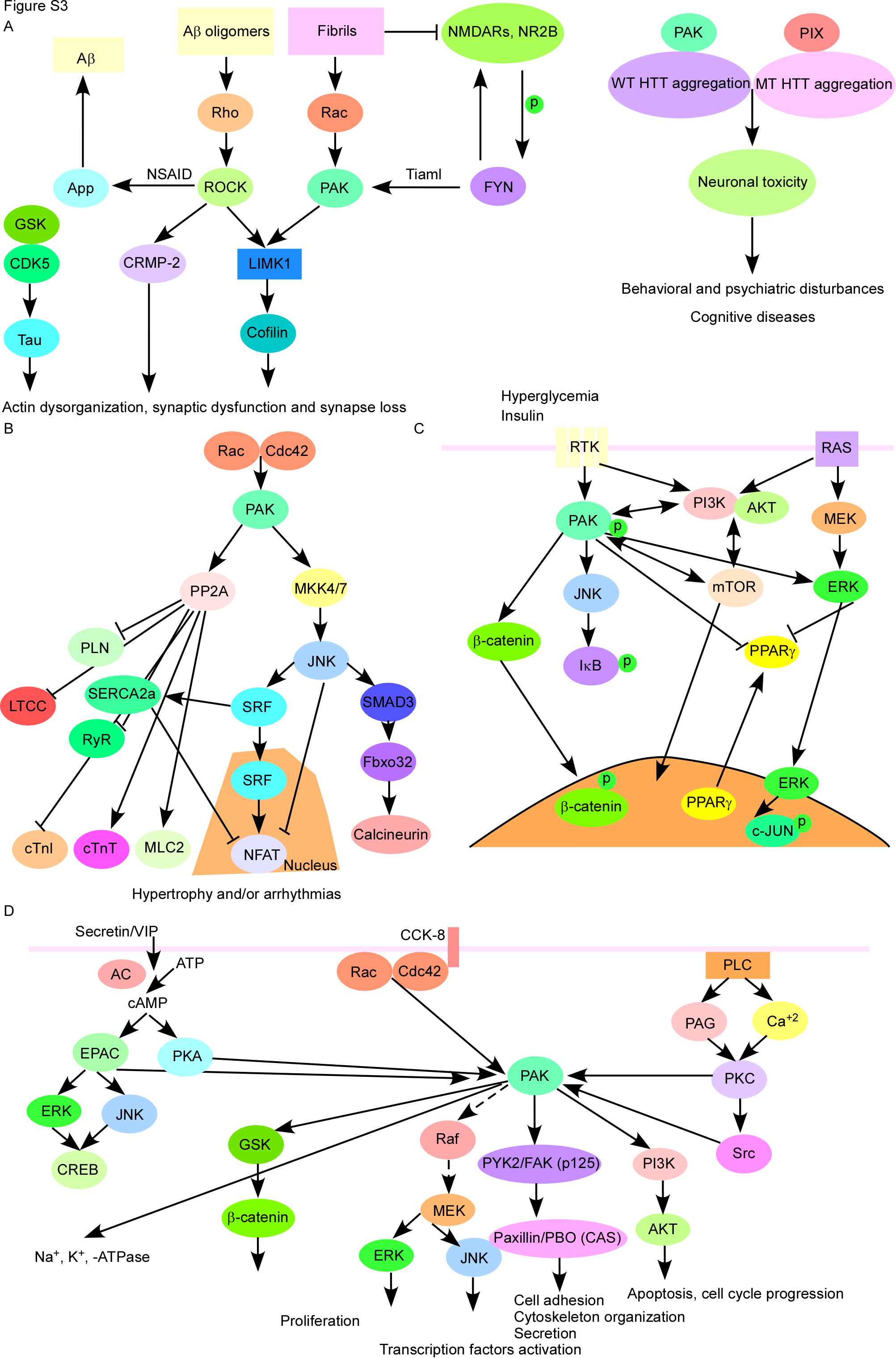

Supplement: Supplementary Figure 3 — PAKs signaling pathways in diseases beyond cancer and infection. (A) PAKs signal transduction in AD (left) and HD (right) pathogenesis. (B) PAKs signaling pathways in heart diseases. (C) PAKs cascades in response to hyperglycemia. (D) PAKs signaling in pancreatic acinar diseases. [file Image_3.TIF]
