# Supplementary material for: The Role of p21-Activated Kinases in Cancer and Beyond: Where Are We Heading?
Source: Front Cell Dev Biol. 2021 Mar 16;9:641381. doi: 10.3389/fcell.2021.641381 (PMC8007885; doi:10.3389/fcell.2021.641381)
Supplement: Supplementary Table 1 — Basic characteristics of PAKs. [file Data_Sheet_1.docx]

Table S1：Basic characteristics of PAKs

| Name | Gene location | Molecular weight (kD) | Tissue expression | Subcellular location | Gene knockout mouse phenotype | References |
| --- | --- | --- | --- | --- | --- | --- |
| PAK1 | 11q13 | 68 | Heart, liver, muscle, spleen | Cytoplasm, nucleus, plasma membrane | Cardiac disorder | (7) |
| PAK2 | 3q29 | 62 | All | Cytoplasm, nucleus, | Embryonically lethal | (8) |
| PAK3 | Xq23 | 65 | Brain | Cytoplasm | Mental retardation | (9) |
| PAK4 | 19q13.2 | 64 | High throughout development, low in adult | Cytoplasm, golgi | Embryonically lethal | (35) |
| PAK5 | 20p12.2 | 81 | Mainly brain, also in adrenal gland, pancreas, testes, prostate | Mitochondria, nucleus, | Abnormal in nervous system | (10) |
| PAK6 | 15q15.1 | 75 | High in testes, prostate, also in brain, kidney, placenta, breast | Cytoplasm, nucleus, | Abnormal skin coloration | (27) |

Table S2 Expression and alteration of PAKs related to human cancers

| Name | Cancer types | Expression and alteration | Pathological features | Prognostic biomarker | References |
| --- | --- | --- | --- | --- | --- |
| PAK1 | Ovarian cancer, breast cancer, colorectal cancer, T-cell lymphoma, hepatocellular carcinoma, renal cancer, bladder cancer, glioblastoma, kidney cancer, squamous cell carcinoma, gestational trophoblastic disease, upper urinary tract cancer, thyroid cancer, oral carcinoma, non-small cell lung cancer, gastric cancer, melanoma, prostate cancer, endometrial cancer, gastroesophageal junction adenocarcinoma, pancreatic cancer, head and neck cancer, esophageal small cell carcinoma | Overexpression  Amplification  Hyperactivation | Tumor grade, tumor progression, invasion, migration, and metastasis, poor outcome and poor prognosis, tamoxifen resistance, 5-fluorouracil resistance | Ovarian cancer, glioma,  colorectal cancer, gastric cancer, head and neck cancer, esophageal small cell carcinoma | (129) |
| PAK2 | Breast cancer, hepatocellular carcinoma, ovarian cancer, lung cancer, gastric cancer | Overexpression, Hyperactivation | Tumor progression, migration  poor prognosis, chemotherapeutic resistance | Pancreatic cancer, prostate cancer, gastric cancer | (129) |
| PAK3 | Thymic neuroendocrine tumor, pancreatic cancer, ovarian cancer | Overexpression | Invasion and migration |  | (63) |
| PAK4 | Pancreatic cancer, oral cancer, ovarian cancer, choriocarcinoma, prostate cancer, squamous cell carcinoma, gallbladder, hepatocellular carcinoma, gastric cancer, glioma, endometrial cancer, breast cancer, colorectal cancer, non-small cell lung cancer, cervical cancer | Overexpression  Amplification | Tumor progression, invasion, migration, and metastasis, poor prognosis, anoikis resistance, cisplatin resistance | Renal cancer, prostate cancer, oral cancer, ovarian cancer.  non-small cell lung cancer | (64) |
| PAK5 | Gastric cancer, hepatocellular carcinoma, non-small cell lung cancer, ovarian cancer, osteosarcoma, esophageal cancer, glioma, colorectal cancer, breast cancer | Overexpression  Gain of function mutations | Tumor progression, paclitaxel resistance, cisplatin resistance |  | (65) |
| PAK6 | Prostate cancer, hepatocellular carcinoma, clear cell renal cell cancer, colon cancer, breast cancer | Overexpression | Tumor progression, poor prognosis, 5-fluorouracil resistance | Clear cell renal cell cancer | (42) |

Table S3 PAKs associated pathogens and related biological process as well as signaling pathways

| Name | Type | PAKs | Mediator | Pathways | Processes | References |
| --- | --- | --- | --- | --- | --- | --- |
| SARS-CoV-2 | RNA | PAK1 | ACE2, TMPRSS2 | MAPK, AP-1, CCL2 | Adaptive immune response, lung fibrosis | (12, 14) |
| HBV | DNA | PAK1 | HBx | PAK1 overexpression | Survival | (87) |
| HCV | RNA | PAK1 | p70 S6 kinase | PI3K, ERK | HCV RNA replication | (66) |
| HHV-8 | DNA | PAK1, PAK2 | GPCR | Rac1/Cdc42, IKKβ, IκB, NF-κB, VE-cadherin | Survival, cellular transformation to kaposi’s sarcoma | (88) |
| HIV | RNA | PAK1,PAK2 | Nef, gp120 | PI3K, BAD, Rac1, LIMK1 | Survival, invasion | (89, 90) |
| HSV | DNA | PAK1 | Nef | Rac1/Cdc42 | Actin stress fiber disassembly, spread of virus | (91) |
| A-MLV | RNA | PAK1 |  | Rac1, RhoG | Increase macropinocytosis | (87) |
| ASFV | DNA | PAK1 | EGFR | Rac1, PI3K/AKT | Entry and infection | (87) |
| VV | DNA | PAK1 | MARCO, phosphotidylserine receptor | Rac1/Cdc42 | Actin rearrangement formation | (92) |
| SGIV | DNA | PAK1 |  | Rac1 | Increased SGIV uptake | (87) |
| HAdV-3 | DNA | PAK1 | CD46, integrins, CtBP1 | Rac1 | Increase macropinocytosis | (87) |
| Influenza A | RNA | PAK1 |  | MEK/ERK | High viral titres | (87) |
| EV1 | RNA | PAK1 | α2β1 integrin | Rac1, PI3K, PLC, PKCα | Macropinosome closure | (87) |
| Alphaherpes-virus | DNA | PAK1, PAK2 | US3 | BAD, Bid, Cofilin | Survival, invasion | (87) |
| ZEBOV | RNA | PAK1 | C-type lectin phosphotidylserine receptor | CtBP/BARS | Increase macropinocytosis | (87) |
| *H.pylori* | Bacteria | PAK1 | T4SS effector, CagA | NIK, IKKs, NF-κB, Rac1 | Inflammatory responses | (96, 97) |
| *E.coli K1* | Bacteria | PAK1 |  | MLCK | Actin mediated internalisation | (87) |
| *S.Typhimurium* | Bacteria | PAK1 | T3SS effectors, SopB, SopE, SopE2 | NF-κB | Pathogen intracellular growth | (93) |
| *EPEC* | Bacteria | PAK1, PAK2 | T3SS effectors, EspG | ARF6 | Reprograms cellular events | (87) |
| *N.gonorrhoeae* | Bacteria | PAK1, PAK2 |  | AP-1, JNK, Rac1/Cdc42 | Inflammatory responses | (87) |
| *NTHi* | Bacteria | PAK1 |  | Rac1, PI3K, Op18/Stathmin | Microtubule polymerization | (87) |
| *B.bacilliformis* | Bacteria | PAK1 |  | Rac1/Cdc42 | Cytoskeleton changes, formation of filopodia and lamellipodia | (87) |
| *T.annulate* | Parasite | PAK1 |  | IKK, IκB, NF-κB | Host cellular transformation, host nuclear responses | (87) |
| *T.cruzi* | Parasite | PAK1 | PDNF | Rac1, AKT | Pathogen survival | (87) |
| *P.falcipaium, P.berghei* | Parasite | PAK1 |  | MEK1 | Pathogen development | (87) |

Abbreviations: HBV: human hepatitis B virus; HCV: hepatitis C virus; HHV-8: human herpes virus 8; HIV: human immunodeficiency virus; HSV: herpes simplex virus; A-MLV: Amphotropic murine leukemia virus; ASFV: African swine fever virus; VV: vaccinia virus; SGIV: Singapore grouper Irido virus; HAdv-3: human adenovirus 3; EV1: nechovirus1; ZEBOV: Zaire ebola virus; EPEC: enteropathogenic *E.coli*; NTHi: non-typeable H.influenza.

Table S4 Inhibitor of PAKs

| Name | Type | Target | Function | References |
| --- | --- | --- | --- | --- |
| KTD606 | ATP competitive | PAK1 | Proliferation | (82) |
| FL172 | ATP competitive | Pan-PAK | Kinase activity | (129) |
| FRAX486 | ATP competitive | Group 1 | Cell growth | (82) |
| AZ-PAK-36 | ATP competitive | Group 1 | Kinase activity | (129) |
| FRAX597 | ATP competitive | PAK1 | Tumorigenesis | (82) |
| K-252a | ATP competitive | PAK1 | Proliferation | (82) |
| R-1 | ATP competitive | PAK1 | Kinase activity | (82) |
| ZMF-10 | ATP competitive | PAK1 | Proliferation | (82) |
| PF-3758309 | ATP competitive | PAK1, PAK4 | Proliferation, Tumor growth | (129) |
| AK963 | ATP competitive | PAK1 | Proliferation | (82) |
| II-11 | ATP competitive | PAK1 | Kinase activity | (82) |
| OSU-03012 | ATP competitive | PAK1 | Cell migration | (82) |
| G5555 | ATP competitive | PAK1 | Kinase activity | (131) |
| G9791 | ATP competitive | PAK1 | Kinase activity | (131) |
| IPA-3 | Allosteric | PAK1 | Inhibit PAK1 related signaling | (82) |
| 2-Mc-1, 4-NHQ | Allosteric | PAK1 | Kinase activity | (82) |
| PP1 | ATP competitive | PAK1 | Inhibit malignant transformation | (129) |
| hPP1 | ATP competitive | PAK1 | Negative PAK regulator | (129) |
| CRIPak | ATP competitive | PAK1 | PAK1 mediated ER transactivation modulation | (129) |
| FRAX1036 | ATP competitive | PAK1, PAK2 | Induce apoptosis | (70) |
| PQA-18 | ATP competitive | PAK2 | Attenuates cytokines, macrophages | (82) |
| KPT-9274 | ATP competitive | PAK4, NAMPT | PhaseⅠ | (132) |
| CZh-226 | ATP competitive | PAK4 | Kinase activity | (82) |
| Prodrug 19 | ATP competitive | PAK4 | Kinase activity | (129) |
| PB-10 | ATP competitive | PAK4 | Proliferation, migration, invasion, and adhesion | (125) |
| 17 | ATP competitive | PAK1, PAK4 | Kinase activity | (82) |
| KY-04031 | ATP competitive | PAK4 | Kinase activity | (126) |
| A2, B6, B8 | ATP competitive | PAK4 | Proliferation | (127) |
| LCH-7749944 | ATP competitive | PAK4, PAK1, PAK5,PAK6 | Proliferation and invasion | (129) |
| KPT-7523 | Allosteric | PAK4 | Proliferation, tumor growth | (128) |
| KY-04031 | ATP competitive | PAK4 | Growth and invasion | (82) |
| 1-phenanthryl-tetrahydroisoquinoline | ATP competitive | PAK4 | Proliferation | (82) |
| Curcumin | Natural | PAK1 | PAK1-LKB1-AMPK, Proliferation and invasion | (11) |
| Propolis | Natural | PAK1 | Hsp16, PAK1-LKB1-AMPK, cancer, inflammatory diseases, MD, T2DB, HIV, HPV, Influenza virus, COVID-19 | (14) |
| Melatonin | Natural | PAK1 | Intracellular adhesion, cancer, immune stimulative, anti-infection, anti-inflammation | (11) |
| Staurosporine | ATP competitive | Pan-PAK | Cancer, COVID-19 | (14) |
| Glaucarubinone | Allosteric | PAK1, PAK4 | AP-1, AKT, Tumor growth, Cancer, COVID-19 | (14) |
| Derivatives of hispidin and mimosine | Natural | PAK1 | Inhibit PAK1 activity | (11) |
| Myricetin | Natural | PAK1 | Induce apoptosis | (11) |
| β-elemene | Natural | PAK1 | Radiosensitivity | (11) |
| Berberine | Natural | PAK1 | Rac/Cdc42, cancer, anti-infection, T2DB, Obesity | (11) |
| Andrographolides | Natural | PAK1 | PAK1-PI3K-AKT, Malaria, COVID-19 | (14) |
| Mesalamine | Natural | PAK1 | Ulcer, cancer, anti-infection, anti-inflammation, T2DB, AD | (11) |
| Bio30 | Mesalamine derivate | PAK1 | Rac1, CDK4, CCND1, cancer | (14) |
| Triptolide | Natural | PAK1 | Cancer, COVID-19 | (14) |
| Minnelide | Natural | PAK1 | AMPK, FOXO, cancer | (11) |
| Salidroside | Natural | PAK1 | Cancer, angiogenesis, osteoporosis, anti-inflammation | (11) |
| MenaQ7 | Salidroside derivate | PAK1 | PAK1-AKT, p21, p27, cancer | (11) |
| Resveratrol | Natural | PAK1 | Cancer | (11) |
| Piceatannol | Natural | PAK1, PAK2 | SYK, PAK-PI3K-AKT, neuronal glucose uptake and insulin sensitivity | (11) |
| PTE | Natural | PAK1 | Cancer, AD | (14) |
| AG490 | PTE derivate | PAK1 | JAK2, LKB1-AMPK, cancer | (11) |
| Capsaicin and capsiate | Natural | PAK1 | Rac1, PAK-PI3K-AKT, LKB1-AMPK, cancer | (11) |
| Sichuan pepercorns | Natural | PAK1 | Cancer, COVID-19 | (14) |
| Ciclesonide | Natural | PAK1 | Anti-inflammation, COVID-19 | (14) |
| Ivermectin from soil bacteria | Natural | PAK1 | Cancer, COVID-19 | (14) |
| Artemisinin | Natural | PAK1 | Ras, Raf, p21, anti-malaria, anti-viral, COVID-19 | (14) |
| Istodax (FK228) | Natural | PAK1 | HDAC-PAK1, COVID-19, FDA approved | (14) |
| Chloroquine (CQ) | Synthetic | PAK1 | P21, anti-malaria, SARS, COVID-19 | (14) |
| Hydroxychloroquine (HQ) | Synthetic | PAK1 | Anti-malaria, COVID-19 | (14) |
| Ketordac | Natural | PAK1 | Rac1, cancer, COVID-19 | (14) |
| 15K | Ketordac derivate | PAK1 | Cancer, COVID-19 | (14) |
| Vitamin D3, and MART-10 | Natural | PAK1 | Cancer, COVID-19 | (14) |
| Azithromycin | Natural | PAK1 | VEGF-PAK1, Angiogenesis, anti-malaria, COVID-19 | (14) |

Table S5 Clinical trials of PAKs

| PAKs | Trial number | Title | Condition | Intervention | Phase | Study type | URL |
| --- | --- | --- | --- | --- | --- | --- | --- |
| PAK1 | NCT04480593 | The Use of Brazilian Green Propolis Extract (EPP-AF) in Patients Affected by COVID-19 | Covid19 | Brazilian Green Propolis Extract (EPP-AF) | Phase 2  Phase 3 | Interventional | <https://ClinicalTrials.gov/show/NCT04480593> |
| PAK3 | NCT00616967 | Carboplatin and Nab-Paclitaxel With or Without Vorinostat in Treating Women With Newly Diagnosed Operable Breast Cancer | Breast Cancer | Carboplatin, paclitaxel albumin-stabilized nanoparticle formulation, vorinostat, placebo | Phase 2 | Interventional | <https://ClinicalTrials.gov/show/NCT00616967> |
| PAK4 | NCT04281420 | A Study of Evaluating Dual Inhibitor of PAK4 and NAMPT ATG-019 in Advanced Solid Tumors or Non-Hodgkin's Lymphoma | Solid Tumor, Non-Hodgkin's Lymphoma | ATG-019, ATG-019 + Niacin ER | Phase 1 | Interventional | <https://ClinicalTrials.gov/show/NCT04281420> |
| PAK4 | NCT02702492 | PAK4 and NAMPT in Patients With Solid Malignancies or NHL (PANAMA) | Solid Tumors, Non-Hodgkin's Lymphoma | KPT-9274, Niacin ER, Nivolumab | Phase 1 | Interventional | <https://ClinicalTrials.gov/show/NCT02702492> |
| PAK4 | NCT00932126 | This Is The First Study Using Escalating Doses Of PF-03758309, An Oral Compound, In Patients With Advanced Solid Tumors | Advanced Solid Tumors | PF-03758309 | Phase 1 | Interventional | <https://ClinicalTrials.gov/show/NCT00932126> |

Table S6 Clinical trials of PAK regulators and effectors

| Name | Trial number | Title | Condition | Intervention | Phase | Study type | URL |
| --- | --- | --- | --- | --- | --- | --- | --- |
| Rac1 | NCT03325088 | Assessing the Expression and the Activity of Rac1 Protein in the Airway Smooth Muscle of Asthmatic Patient | Asthma | Bronchial  Endoscopy,  Bronchial biopsies,  Bronchial alveolar  enema |  | Interventional | <https://ClinicalTrials.gov/show/NCT01693549> |
| Rac1 | NCT02450058 | Adjuvant FEC Versus EP in Breast Cancer (MIG5) | Breast Cancer, Chemotherapy, Adjuvant | 5-fluorouracil,  Epirubicin,  Cyclophosphamide,  Paclitaxel | Phase 3 | Interventional | <https://ClinicalTrials.gov/show/NCT02450058> |
| Rac1 | NCT00990639 | Effect of Candesartan in Alcoholic Liver Fibrosis | Alcoholic Liver Disease | Candesartan for hepatic fibrosis | Phase 1  Phase 2 | Interventional | <https://ClinicalTrials.gov/show/NCT00990639> |
| Rac1 | NCT02862886 | Study of the Effect of Beta-3 Adrenergic Receptor Agonists on Oxidative Stress and Signalling Pathways Implicated in Remodelling of the Uteroplacental Space: Experimental in Vitro Approach on Myometrial Tissue Explants | Pregnancy | Myometrium biopsy |  | Interventional | <https://ClinicalTrials.gov/show/NCT02862886> |
| Rac1 | NCT00005581 | Combination Chemotherapy in Treating Women With Stage II or Stage III Breast Cancer | Breast Cancer | Cyclophosphamide,  Epirubicin hydrochloride,  Fluorouracil,  Paclitaxel | Phase 3 | Interventional | <https://ClinicalTrials.gov/show/NCT00005581> |
| Rac1 | NCT04235621 | A Study to Understand the Genetics and Clinical Course of Focal Segmental Glomerulosclerosis (FSGS), Treatment-Resistant Minimal Change Disease (TR-MCD), and Diabetic Nephropathy (DN) | Glomerulosclerosis, Focal Segmental,  Minimal Change Disease,  Diabetic Nephropathies | FSGS/TR-MCD,  Diabetic Nephropathy (DN) |  | Observational | <https://ClinicalTrials.gov/show/NCT04235621> |
| Rac1 | NCT03088878 | A Study of Cirmtuzumab and Ibrutinib in Patients With B-Cell Lymphoid Malignancies | B-cell Chronic Lymphocytic Leukemia,  Small Lymphocytic Lymphoma,Mantle Cell Lymphoma |  | Phase 1  Phase 2 | Interventional | <https://ClinicalTrials.gov/show/NCT03088878> |
| LIMK | NCT01693549 | Study of Cabazitaxel in Patients With Metastatic Breast Cancer Previously Treated With Taxanes | Breast Cancer | Cabazitaxel | Phase 2 | Interventional | <https://ClinicalTrials.gov/show/NCT01693549> |
| Merlin | NCT03192306 | Safety and Efficacy of Merlin (Ethanol and Glycolic Acid Mixture) for Episodic Treatment of Cold Sores | Recurrent Herpes Labialis | Merlin,Ethanol | Phase 2 | Interventional | <https://ClinicalTrials.gov/show/NCT03192306> |
| Merlin | NCT01985321 | Safety and Efficacy of Merlin (Ethanol and Glycolic Acid Mixture) for the Treatment of Cold Sores | Recurrent Herpes Labialis | Ethanol/glycolic acid solution,  Placebo/Ethanol | Phase 2 | Interventional | <https://ClinicalTrials.gov/show/NCT01985321> |
| Merlin | NCT04589585 | Clinical Study of Merlin's DiVeRt - Vascular Reconstruction Device and Delivery System | Intracranial Aneurysm, Wide Neck Aneurysm, Large Neck Aneurysm, Lesion |  |  | Interventional | <https://ClinicalTrials.gov/show/NCT04589585> |
| Merlin | NCT04702997 | A Trial of Bardoxolone Methyl in Patients With CKD at Risk of Rapid Progression (MERLIN) | Chronic Kidney Diseases | Bardoxolone methyl oral capsule,  Placebo oral capsule | Phase 2 | Interventional | <https://ClinicalTrials.gov/show/NCT04702997> |
| Merlin | NCT03710564 | Study of Safety and Efficacy of Brolucizumab 6 mg Dosed Every 4 Weeks Compared to Aflibercept 2 mg Dosed Every 4 Weeks in Patients With Retinal Fluid Despite Frequent Anti-VEGF Injections | Age-Related Macular Degeneration | Brolucizumab,  Aflibercept | Phase 3 | Interventional | <https://ClinicalTrials.gov/show/NCT03710564> |
| Merlin | NCT02997878 | A Single-arm,Phase IIa,Safety and Efficacy Trial of Selected MSCs in the Treatment of Patients With PSC & AiH | Cholangitis, Sclerosing,Hepatitis, Autoimmune | Orbcel-C | Phase 1，Phase 2 | Interventional | <https://ClinicalTrials.gov/show/NCT02997878> |
| Merlin | NCT01024946 | Everolimus (RAD001) for the Treatment of Malignant Pleural Mesothelioma With Merlin/NF2 Loss as a Biomarker to Predict Sensitivity | Malignant Pleural Mesothelioma | Everolimus | Phase 2 | Interventional | <https://ClinicalTrials.gov/show/NCT01024946> |
| Merlin | NCT04665206 | Study to Evaluate VT3989 in Patients With Metastatic Solid Tumors Enriched for Tumors With NF2 Gene Mutations | Solid Tumor, Adult,  Mesothelioma | VT3989 | Phase 1 | Interventional | <https://ClinicalTrials.gov/show/NCT04665206> |
| Merlin | NCT01870609 | Placebo Controlled Study of VS-6063 in Subjects With Malignant Pleural Mesothelioma | Malignant Pleural Mesothelioma | Defactinib (VS-6063),  Placebo | Phase 2 | Interventional | <https://ClinicalTrials.gov/show/NCT01870609> |
| Merlin | NCT02523014 | Vismodegib and FAK Inhibitor GSK2256098 in Treating Patients With Progressive Meningiomas | Intracranial Meningioma,  Recurrent Meningioma,NF2 Gene Mutation | vismodegib,  GSK2256098 | Phase 2 | Interventional | <https://ClinicalTrials.gov/show/NCT02523014> |
| Merlin | NCT00340496 | Analysis of NF2 Mutations in Radiation-Related Neural Tumors | Neural Tumors |  |  | Observational | <https://ClinicalTrials.gov/show/NCT00340496> |
| Merlin | NCT00932893 | An Investigational Drug, PF-02341066 Is Being Studied Versus Standard Of Care In Patients With Advanced Non-Small Cell Lung Cancer With A Specific Gene Profile Involving The Anaplastic Lymphoma Kinase (ALK) Gene | Carcinoma, Non-Small-Cell Lung | PF-02341066,  Pemetrexed,  Docetaxel | Phase 3 | Interventional | <https://ClinicalTrials.gov/show/NCT00932893> |
| Merlin | NCT00932451 | An Investigational Drug, PF-02341066, Is Being Studied In Patients With Advanced Non-Small Cell Lung Cancer With A Specific Gene Profile Involving The Anaplastic Lymphoma Kinase (ALK) Gene | Carcinoma, Non-Small-Cell Lung | PF-02341066 | Phase 2 | Interventional | <https://ClinicalTrials.gov/show/NCT00932451> |
